# Health system efficiency in OECD countries: dynamic network DEA approach

**DOI:** 10.1186/s13561-021-00337-9

**Published:** 2021-10-12

**Authors:** Beata Gavurova, Kristina Kocisova, Jakub Sopko

**Affiliations:** 1 Center for Applied Economic Research, Faculty of Management and Economics, Tomas Bata University in Zlín, Mostní 5139, 760 00 Zlín, Czech Republic; 2 Faculty of Economics, Technical University of Košice, Němcovej 32, 040 01 Košice, Slovak Republic

**Keywords:** Public health, Data envelopment analysis, DNDEA, OECD, Efficiency, C61, I11, R11, R58

## Abstract

**Background:**

In recent years, measuring and evaluating the efficiency of health systems has been explored in the context of seeking resources to ensure the sustainability of ‘countries’ health and social systems and addressing various crises in the health sector. The study aims to quantify and compare the efficiency of OECD health systems in 2000, 2008, and 2016. The contribution to research in the field of efficiency in the healthcare system can be seen in the application of Dynamic Network Data Envelopment Analysis (DNDEA), which help us to analyse not only the overall efficiency of the healthcare system but analyse the overall efficiency as the result of the efficiencies of individual interconnected areas (public and medical care area). By applying the DNDEA model, we can realise the analysis not only within one year, but we can find out if the measures and improvements taken in the healthcare sector have a positive impact on its efficiency in a later period (eight-year interval).

**Methods:**

The analysis focuses on assessing the efficiency of the health systems of OECD countries over three periods: 2000, 2008, and 2016. Data for this study were derived from the existing OECD database, which provides aggregated data on OECD countries on a comparable basis. In this way, it was possible to compare different countries whose national health statistics may have their characteristics. The input-oriented Dynamic Network Data Envelopment Analysis model was used for data processing. The efficiency of OECD health systems has been analysed and evaluated comprehensively and also separately in two divisions: public health sub-division and medical care sub-division. The analysis combines the application of conventional and unconventional methods of measuring efficiency in the health sector.

**Results:**

The results for the public health sub-division, medical care sub-division and overall health system for OECD countries under the assumption of constant returns to scale indicate that the average overall efficiency was 0.8801 in 2000, 0.8807 in 2008 and 0.8472 in 2016. The results of the input-oriented model with the assumption of constant returns to scale point to the overall average efficiency of health systems at the level of 0.8693 during the period. According to the Malmquist Index results, the OECD countries improved the efficiency over the years, with performance improvements of 19% in the public health division and 8% in the medical care division.

**Conclusions:**

The results of the study are beneficial for health policymakers to assess and compare health systems in countries and to develop strategic national and regional health plans. Similarly, the result will support the development of international benchmarks in this area. The issue of health efficiency is an intriguing one that could be usefully explored in further research. A greater focus on combining non-parametric and parametric models could produce interesting findings for further research. The consistency in the publication and updating of the data on health statistics would help us establish a greater degree of accuracy.

## Introduction

In the last three decades, many significant changes in the health sector, mainly affected by the global ageing process, were evident. It impacts the present state of health systems in the individual countries and their sustainability. The international organisations, such as the WHO, OECD, Eurostat and others, also play a partial role. These organisations emphasise the quality and availability of health services and a fair way of financing and investments in this field. Healthcare is one of the areas of the public sector where more funding does not automatically mean better results or improvement of the population’s health condition [1]. The use of optimal measurement systems will not only measure and evaluate the efficiency of individual interventions in health systems and relevant policies, but it will also create benchmarking indicators for comparing health systems across multiple countries. To maximise the positive impact, effective health management needs to consider several aspects such as financing, the healthcare market, structure and regulatory stabilisation mechanisms. The present upwards trend of computerisation of society and technological progress only highlights the importance of improving performance measurement in healthcare. The main concern of health policymakers and managers are the efforts to measure efficiency [2], with many countries having introduced reforms to improve health systems over the past three decades [3, 4]. Several studies conclude that the main issue in all health systems is inefficiency [5–8]. WHO [6] claims that there are evident global differences in the efficiency of health systems. The main issue is in finding optimal methods to measure health efficiency and how they may apply to good policy or management decisions.

Measurement of health efficiency is often very complicated due to external factors, including social determinants of health. Many of these factors cannot be affected by the health sector. Therefore, any efficiency measure in this area must take into account the impact of external factors. Cylus et al. [9] state that there are effectively functioning components within an inefficient health system. An example is the hospital sector and/or parts thereof. While the authors remind us that the hospital sector may be efficient, it is very likely that hospitals can also operate in an extremely inefficient health system. In this area, preventive health measures may not receive sufficient attention or lack of primary health care. Therefore, we emphasise the importance of monitoring several health system levels to determine the nature and extent of inefficiency.

Measuring health efficiency is a challenging process that involves two groups of risks: scepticism in identifying and finding ways to eliminate the inefficiency of the health system and improperly set strategies that may inefficiently reduce spending in high-efficiency areas of the system. Efficiency, not only in healthcare but also in the economy, is important for national leaders to set financial stability, long-term economic sustainability, and increase competitiveness.

Generally, there are many interrelated processes in the health system that may be evaluated independently. To ensure best practice, it is possible to determine whether these processes are effective or ineffective. A large number of indicators are monitored in the health system to identify and quantify inefficiency. However, the interpretation of many of them in a comprehensive assessment of health efficiency may be quite questionable.

It is hoped that this work will lead to new insights of further development of methods for assessing efficiency and examining their limitations, not only from their process but also from the perspective of outputs and their implementation in relevant policies. This consistent fact has also prompted this research to examine the efficiency of health systems in OECD countries during 2000, 2008 and 2016 in two areas: public health and medical care. The findings of the analyses will provide a valuable platform for national health policymakers and support the development of international and national benchmarks in this field.

## Literature review

The sustainable pressure on the proper use of resources in the health sector has led the main actors and policymakers to look for efficient ways of delivering health services. Any improvement in the health system, although incrementally, may bring significant savings in financial resources. Effective healthcare should be a priority for governments in all countries. The analysis of the efficiency of health systems compared to other sectors is much more complicated due to the heterogeneity of health processes, the provision of health services and the determinants of the healthcare market in all countries. Several methods are used to assess the efficiency of health systems in research studies, while Data Envelopment Analysis (DEA) and Stochastic Frontier Analysis (SFA) have been widely reported and extensively explored in the past three decades [10].

Ozcan [5] has shown that classical comparative methods and models based on ratios bring more complications than solutions. According to the author, the evaluation of organisational unit efficiency should be based on optimisation techniques and their normative structure while providing reference criteria and a way to improve the efficiency of lagging organisational units. Each organisational unit requires an individual form of efficiency assessment. The optimal combination of inputs and outputs plays an important role (e.g. [11]). Regarding efficiency, the quality of provided healthcare is also linked, and a relationship between these two categories is examined in several dimensions.

Singaroyan et al. [12] concluded that improving healthcare quality does not always lead to effective processes. Mobley & Magnussen [13] have shown that poor performance in healthcare is due to low efficiency. Helling et al. [14] confirmed that increasing the efficiency rate also increases the quality of provided healthcare. There exists a very extensive literature on the topic of the ability of individual health systems to transform resources into desired outcomes. However, their common feature is identifying areas that produce inefficiency in health systems, leading to excessive spending on health.

A common feature of these studies is applying the DEA to estimate the relative technical efficiency of health systems. Retzlaff-Roberts et al. [15] used an input- and output-oriented model with the assumption of a variable returns to scale (VRS). Bhat [16] used an input-oriented DEA model based on the assumption of the constant returns to scale (CRS). Adang & Borm [17] performed the output-oriented DEA using CRS model and supported the analysis with results of the Malmquist Index. Li et al. [18] calculated the efficiency score based on an input-oriented DEA model with CRS in a specific group of transition economies, comparing the results with the OECD countries. Zeng et al. [19] applied an output-oriented DEA model with the specific weight boundaries for outputs to calculate the technical efficiency of individual HIV/AIDS prevention programs. Medeiros & Schwierz [20] used the DEA model with the VRS based on the work of Hollingsworth & Smith [10]. They also performed the SFA and bootstrapping approaches in their analysis. Li et al. [18] conclude that initially, the research of health ‘systems’ efficiency measurement was focused more on an organisational or corporate level (e.g. hospitals or health institutions; Kohl et al. [21] in their extensive study investigated 262 scientific articles in this area).

Drawing on an extensive range of sources, these studies set out similar ways to select inputs and outputs. With this in mind, we describe selected variables, which are also used in our analysis. On the input side, the per capita health expenditure (in US$ in PPP, or expressed as a per cent of GDP), the number of physicians per 1000 inhabitants or tobacco consumption are often used. The importance of selecting the indicator of health expenditure in measuring the efficiency in the health sector is also investigated by Blendon et al. [22, 23]. The number of physicians per 1000 people and its importance is also commented on by Grubaugh & Santerre [24]. Contoyannis & Jones [25] and Shaw et al. [26] have also reported tobacco consumption as an important indicator in the health field. However, there is a great deal of debate surrounding alcohol consumption (measured in litres per capita) involving this indicator in the analysis. For instance, Conotyannis & Jones [25] have not shown a significant effect of alcohol consumption on life expectancy at birth (LE), neither Adang & Borm [17], Retzlaff-Roberts et al. [15].

Nevertheless, Li et al. [18] include this indicator in their analyses. On the other side, Bhat [16] demonstrated by Spearman’s correlation coefficient that alcohol and tobacco consumption impact the level of health. The outputs are life expectancy at birth, infant mortality rate, and/or avoidable mortality in several studies.

The causes of missing data have been the subject of intense debate within the scientific community. Grubaugh & Santerre [24] recommend using a smaller sample of countries for comparison and not including the country with missing data in the analysis. On the other hand, Anderson et al. [27], indicate a possibility of using data that are the closest to a reference year or a period for which the analysis is realised. As mentioned above, an important part of health efficiency analysis is the proper selection of inputs and outputs and subsequent estimation of the relative efficiency of health systems, with a high emphasis on a correct interpretation of results.

In the work of Afonso & Aubyn [28], the DEA and the Free Disposal Hull (FDH) approaches are used to estimate health and education efficiency in 24 OECD countries. Luoma & Räty [29] give a critical view of the study, pointing out the differences that may be created in efficiency by using more appropriate forms of inputs and outputs. They criticise an interpretation of the results of the input and output-oriented DEA model with the VRS. Afonso & Aubyn [28] used one input and one output to estimate efficiency. It may lead to an overestimation of the results for eight OECD countries in the output-oriented model. Luoma & Räty [29] performed the same efficiency estimation process for each country. However, the authors used revised data and excluded the number of hospital beds from inputs as they do not consider this indicator to be relevant input in the analysis. Similarly, the authors have considered the aggregate indicator of the population’s health status and life expectancy at birth. Suppose this indicator is used as an output. In that case, they do not recommend including in the analysis of health systems such inputs that only refer to employees (e.g. number of physicians and nurses) and technical aspects of institutions (e.g. the number of beds). The main reason is that the indicator of life expectancy at birth depends on a certain standard of the population’s lifestyle and environmental and cultural factors. Similar work has also been pursued by Spinks & Hollingsworth [30] in which they discussed the use of life expectancy at birth. The authors have considered a well-organised data collection, which takes into account a quality of life (e.g. quality-adjusted life years (QALYs) or health-adjusted life expectancy (HALE)). Medeiros & Schwierz [20] only confirmed that efficiency measurements might be performed in two ways. On the one hand, by increasing health outcomes while maintaining the current inputs (output-oriented models). On the other, by reducing inputs at the current health outcomes (input-oriented models).

Traditional DEA models are used in many works, and these models provide a basis for measuring efficiency based on inputs and outputs. These conventional DEA models do not distinguish the efficiency of the various components and/or health system. Woolf & Aron [31] recommend, in the case of international comparisons of the level of health systems, examining the efficiency between public health and medical care as components of the whole health system. The evidence reviewed here seems to suggest a pertinent role for deeper discussion. Ozcan & Khushalani [32] investigated the efficiency of public health and medical care systems on a sample of 34 OECD countries between 2000 and 2012. This study differs from other studies in methodology, as the authors use a new approach in the DEA analysis. The analysis is based on the Dynamic Network Data Envelopment Analysis, where the authors used a non-oriented model with CRS. This method is backed up by the evidence in Kawaguchi et al. [33]. Ozcan & Khushalani [32] applied the DNDEA model under the CRS. The authors focused on a specific period in which some health reforms were undertaken in OECD countries, with a common feature of reducing costs and increasing value-added for resources.

Most of the mentioned studies used traditional indicators and traditional DEA models to analyse the efficiency of the health sector as a whole (e.g. [2, 9, 11, 15, 17–20, 28, 34–38]). But the development in the last years pointed to the fact that the overall efficiency of the health sector is affected by the efficiency in different interconnected areas (e.g. [32, 33, 39–41]). By analysing the efficiency of the health sector as a whole, we are losing information about activities within the individual areas. Therefore, we decide to apply the network DEA model, which help us to analyse the efficiency in individual areas of the health sector, taking into account links between them. As mentioned by the expert panel on Understanding Cross-National Health Differences Among High-Income Countries, the health system encompasses the entire continuum between public health and medical care area. The public health area represents an area of healthcare where efficiency could be affected by policymakers and experts in health sectors. For example, they can take decisions in the legislative field, which can help reduce alcohol or tobacco consumption, increase vaccination rates against various dangerous diseases, educate people about health issues, and this way increase the overall life expectancy of the population. The medical care area represents way how health services are delivered to patients. By analysing this area of the health system, we can find out if increasing investment into medical technologies or increasing the number of medical staff can help to improve the early diagnosis of diseases and increase the number of hospital discharges. It is essential to say that both areas are interconnected as the decision of policymakers and experts can improve the efficiency not only within the health care area but also within medical care area as it can increase the number of vaccinated persons or a number of cancer screening which help to improve the early diagnosis of diseases. Therefore, we decide to involve these variables as linking between both areas, which can be considered as contribution of our paper compared to traditional DEA models.

Another shortcoming of previous studies is the application of traditional DEA models for each year separately (e.g. [2, 9, 15, 17–20, 28, 34–37]). This unable us to take into account activities which are transmitted between years. We could not consider that applying new technologies or reducing alcohol and tobacco consumption will positively affect life expectancy or infant mortality in the same year. Therefore, we decide to apply a dynamic DEA model that allows us to apply carry-over activities, which can positively affect the efficiency in different areas not in the same, but at a later time. With the combination of both aspects, network and dynamic, we can analyse the development of efficiency in different areas of health sectors in OECD countries between the years 2000, 2008, and 2016. This way, we can apply not only standard inputs and outputs like alcohol and tobacco consumption, life expectancy, and employment but also variables like immunisation, cancer screening, and so on. Through the application of so-called link variables, we can analyse if, for example, the increasing vaccination, increasing number of mammography screenings based on the decision of policymakers and experts in health sectors can improve the efficiency of medical care and can have a positive effect on the life expectancy of people leaving in the analysed countries. We can also investigate if the decreasing infant mortality or decreasing number of new cancer cases in the specified year resulted from a better condition in the health system can have a positive effect on the efficiency in different areas, and this way on the overall efficiency of the health sector in later time (in our sample eight years later).

Compared to the previous studies (e.g. [2, 11, 17–20, 32, 36, 41, 42]), we decide to apply the input-oriented DNDEA model as we want to find out how the country could set its inputs effectively to reach given outputs. We analyse the optimal reduction in alcohol and tobacco consumption in the public health area, leading to given health-adjusted life expectancy in the specified country. In the medical care area, we explore if the medical technologies and employees are used effectively compared to the provided services to patients. In calculating efficiency, we also consider links between both areas and variables that can influence the efficiency with time shift. As seen in the literature review, some authors prefer to use the model under the constant returns to scale assumption (e.g. [2, 10, 16, 17], [18, 32, 37], or [41]), while others prefer variable returns to scale assumption (e.g. [11, 15, 20, 34], or [36]). The advantage of VRS is that we can eliminate size differences between countries. But on the other hand, as mentioned by [10], when variables are expressed in ratio form, we can apply CRS model, as ratio form eliminates size effect. To verify if there are significant differences between both assumptions in the case where ratios are used, we also apply constant and variable returns to scale model, which also can be considered as the contribution of the paper.

Taking these studies into account, we emphasise that the health system consists of several subdivisions. Therefore, this study aims to contribute to this growing area of research by exploring a novel approach to assess health efficiency. Considering all of this evidence, it seems that applying a combination of dynamic and network DEA analysis will serve as a continuous impulse for future research. To fill a gap in the literature, we have decided to apply our investigation not only to EU countries but also to OECD countries, which offers us the possibility of a more extensive comparison of a number of developed economies.

## Methodology and data

The analysis focuses on assessing the efficiency of the health systems of OECD countries over three periods: 2000, 2008 and 2016 using DEA analysis. The DEA, as an analysis based on the application of mathematical programming, was initially specified by Charnes et al. [43] based on the work of Farell [44], and later developed by Banker et al. [45], Debreu [46], Shepherd [47] and Afriat [48]. Since the first introduction of the DEA method in 1978, the DEA has been widely used in many investigational studies to model and evaluate operational process performance, which does not require strict assumptions as in other approaches, and is, therefore, more flexible. This method is currently the subject of further studies and applications within each model [49–53]. The DEA analysis includes two basic conceptual models: the CCR model, named after Charnes, Cooper and Rhodes [43] and the BCC model, first introduced by Banker, Charnes and Cooper [34]. The essence of the difference between these models results from an approach to returns to scale. The CCR model assumes constant returns to scale. The BCC model assumes a VRS. The individual weights for the evaluated decision-making units (DMUs) in the DEA analysis are calculated to maximise unit efficiency. A hypothetical (virtual) unit, characterised as a weighted average of certain actual values of effective units, may be determined for inefficient units. Such a unit (the size of its inputs and outputs) serves as a model for a real inefficient unit that produces fewer outputs or consumes more inputs than its virtual unit. For the VRS models, the requirement that α times the input must be balanced by increasing the output by the same α times, does not apply. Thus, the DMU may be efficient even if a relative increase in outputs will be lower or higher than the increase in inputs [54].

**Fig. 1:**
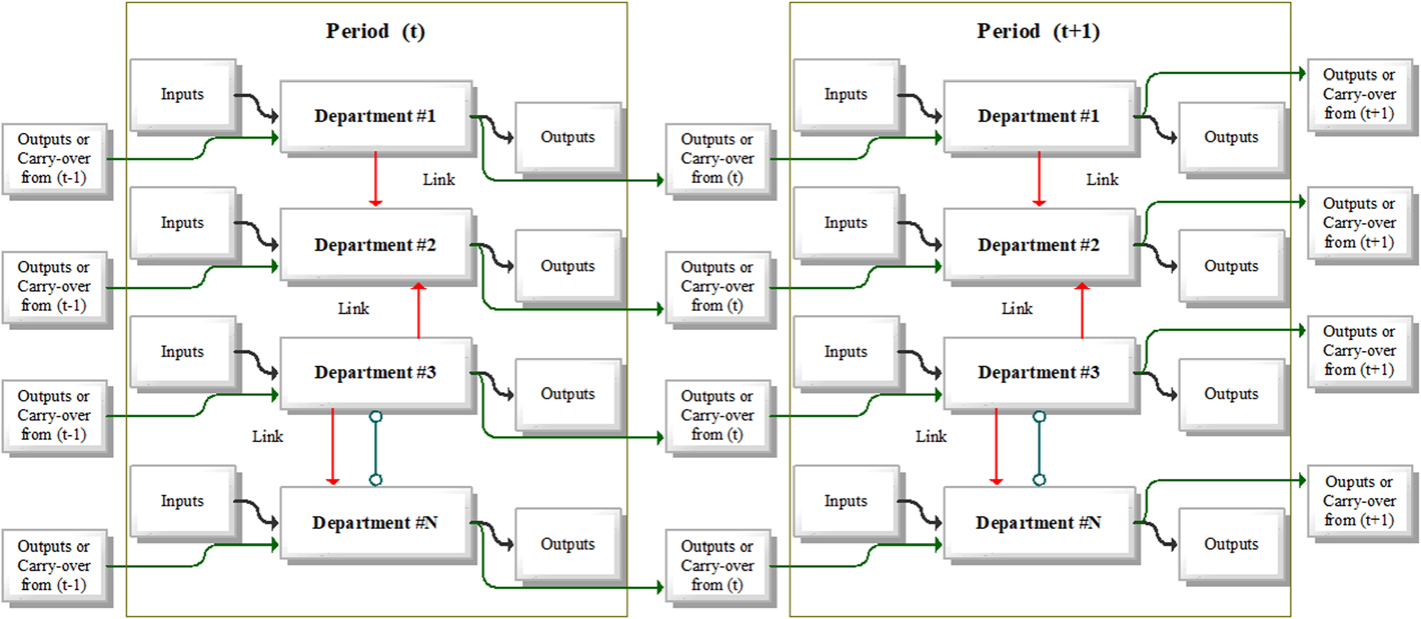
Dynamic and network DNDEA model concept. Source: Prepared by authors based on Ozcan [5]

The Dynamic Network DEA represents the approach taken by other authors to address the efficiency with a combination of dynamic and network DEA analysis [55]. Standard DEA models do not analyse and contain no information about the internal structure between the individual DMUs examined. The efficiency score ranges between 0 and 1, where one indicates the unit is relatively efficient, and a value < 1 indicates inefficiency. In the health sector, the services are provided through several areas (departments, or divisions) that contribute to the overall efficiency of a larger unit (e.g. hospitals). In monitoring the efficiency of hospitals, the entire group includes, apart from individual hospitals, other areas, such as nursing homes, medical offices, outpatient surgical centres and diagnostic centres. Traditional DEA models do not take into account the internal structure of health organisations that act as DMUs. Network DEA models are also reported by Färe & Grosskopf [56] and later by Lewis & Sexton [39], who applied network DEA analysis between teams in the professional Major League Baseball in North America. Tone & Tsutsui [40] expanded these core models by developing a network model using slacks through the work of Tone [57], which is a non-radial model for measuring efficiency in the case of a disproportionate change in inputs and outputs. The basic graphical concept of the internal structure of dynamic and network DEA models may be seen in Fig. 1. Areas (divisions) of the health sector represent a subunit of a healthcare facility, where each area may have its inputs and outputs and may also be linked to other divisions. Dynamic network DEA models identify linking activities while taking into account the internal heterogeneous structure of DMUs.

**Table 1:** Definition of variables used in DNDEA model

Variable	Definition
${x}_{ijk}^t$	An input resource *i* to *DMU*_*j*_ for the sub-division *k* at period *t*
${y}_{rjk}^t$	An output product *r* from *DMU*_*j*_ for the sub-division *k* at period *t*
${z}_{j{(kh)}_l}^t$	A linking intermediate product of *DMU*_*j*_ from sub-division *k* to sub-division *h* at period *t*
${z}_{j{k}_l}^{\left(t,t+1\right)}$	A carry−over of *DMU*_*j*_ at the sub-division *k* from period *t* to period *t* + 1
${s}_{iok}^{t-}$	A slack of the input *i* of *DMU*_*o*_ for sub-division *k* at period *t*
${s}_{rok}^{t+}$	A slack of the output *r* of *DMU*_*o*_ for sub-division *k* at period *t*
${s}_{o{(kh)}_l\alpha}^t$	A slack of link(*kh*)_*l*_ of *DMU*_*o*_ at period *t*. *α* stands for *free*, " *as input* " and " *as output*"
${s}_{ok_l\alpha}^{\left(t,t+1\right)}$	A slack of carry-over variable *k*_*l*_ from period *t* to period *t* + 1. *α* stands for *free*, *good* and *bad*
${\lambda}_{jk}^t$	An intensity of the *DMU*_*j*_ corresponding to sub-division *k* at period *t*
${s}_{ok_l good}^{\left(t,t+1\right)};{s}_{ok_l bad}^{\left(t,t+1\right)};\mathrm{and}\ {s}_{ok_l free}^{\left(t,t+1\right)}$	The slacks denoting, respectively, carry-over shortfall, carry-over excess and carry-over deviation
*ngood*_*k*_; *nbad*_*k*_; *nfree*_*k*_	The number of desirable (good), undesirable (bad) and free carry-over variables for each sub-division *k*.

Source: Prepared by authors

The major advantage of applying the network DEA analysis is the assumption that the overall efficiency of the health system depends not only on political decisions but also on healthcare provision. With this aim in mind, in this paper, the efficiency is examined in two separate areas of the health system, namely the efficiency in the field of public health and the efficiency in medical care.

It is necessary to determine the so-called, linking activities’ variables between divisions within the network DEA analysis. These variables represent activities that may affect the efficiency of the second division, but in terms of their applicability, they are regulated by the first division. An advantage of the dynamic network DEA models is that the efficiency measurement may be performed between periods (time period *t, t + 1*, etc.). Since the DNDEA analysis combines network and dynamic DEA analysis, it is necessary to include the carry-over variables, creating intermediate links between variables from one to the other period. These carry-over variables may affect efficiency in the next period, either positively or negatively. Consequently, good and bad (or positive and negative) carry-over variables are considered. The mathematical formulation of the dynamic network DNDEA analysis mentioned below is based on Lewis & Sexton [39]; Tone & Tsutsui [40], Kawaguchi et al. [33] and Ozcan & Khushalani [32].

DNDEA analysis can be adapted by considering *n DMUs* (*j* = 1, …, *n*), which consist of *K* sub-divisions (*k* = 1, …, *K*) during *T* time periods (*t* = 1, …, *T*). It may be considered that *m*_*k*_ will represent the number of inputs and *r*_*k*_ the number of outputs to sub-division *k*. Links between individual divisions (e.g., from division *k* to division *h*) will be denoted as (*k*, *h*)_*l*_ with the set of such links being denoted as *L*_*kh*_. The observed data are as follows:

$\left\{{x}_{ijk}^t\in {R}_{+}\right\}\left(i=1,\dots, {m}_k;j=1,\dots, n;k=1,..,K;t=1,\dots, T\right)$ is an input resource *i* to the unit *DMU*_*j*_ of the sub-division *k* in the period *t*. Analogically, $\left\{{y}_{rjk}^t\in {R}_{+}\right\}\left(r=1,\dots, {r}_k;j=1,\dots, n;k=1,..,K;t=1,\dots, T\right)$ represents an output product *r* from the unit *DMU*_*j*_ for the sub-division *k* in the period *t*. Also, undesirable outputs are treated as inputs to the division *k*.

$\left\{{z}_{j{(kh)}_l}^t\in {R}_{+}\right\}\left(j=1,\dots, n;l=1,\dots, {L}_{kh};t=1,\dots, T\right)$ represents the linking intermediate products of *DMU*_*j*_ from sub-division *k* to sub-division *h* in period *t*, where *L*_*kh*_ represents the number of items in the links from *k* to *h*.

$\left\{{z}_{j{k}_l}^{\left(t,t+1\right)}\in {R}_{+}\right\}\left(j=1,\dots, n;l=1,\dots, {L}_k;k=1,\dots, K;t=1,\dots, T-1\right)$ represents the carry-over of *DMU*_*j*_, sub-division *k* from period *t* to the period *t* + 1, where *L*_*k*_ is considered as the number of items in the carry-over from sub-division *k*.

The decision-making unit *DMU*_*o*_ (*o* = 1, …, *n*) ∈ *P*^*t*^ can be specified as follows. The input and output constraints can be expressed as follows:

$$ {\mathbf{x}}_{ok}^t={\mathbf{X}}_k^t{\boldsymbol{\uplambda}}_k^t+{\mathbf{s}}_{ko}^{t-}\ \left(\forall k,\forall t\right) $$

$$ {\mathbf{y}}_{ok}^t={\mathbf{Y}}_k^t{\boldsymbol{\uplambda}}_k^t-{\mathbf{s}}_{ko}^{t+}\ \left(\forall k,\forall t\right) $$

$$ \mathbf{e}\ {\boldsymbol{\uplambda}}_k^t=1\ \left(\forall k,\forall t\right) $$

1 $$ {\boldsymbol{\uplambda}}_k^t\ge \mathbf{0},{\mathbf{s}}_{ko}^{t-}\ge \mathbf{0},{\mathbf{s}}_{ko}^{t+}\ge \mathbf{0},\left(\forall k,\forall t\right) $$

where ${\mathbf{X}}_k^t=\left({\mathbf{x}}_{1k}^t,\dots, {\mathbf{x}}_{nk}^t\right)\in {R}^{m_k\times n\times T}$ a ${\mathbf{Y}}_k^t=\left({\mathbf{y}}_{1k}^t,\dots, {\mathbf{y}}_{nk}^t\right)\in {R}^{r_k\times n\times T}$ represent the input and output matrices and ${\mathbf{s}}_{ko}^{t-}\ \mathrm{and}\ {\mathbf{s}}_{ko}^{t+}$ represent, respectively, input and output slacks. Different opinions were put forward regarding the linking constraints. The “as input” link value case, the linking activities are conducted as input to succeeding division and excesses are accounted for in the input inefficiency:

2 $$ {\mathbf{z}}_{o(kh) in}^t={\mathbf{Z}}_{(kh) in}^t{\boldsymbol{\uplambda}}_k^t+{\mathbf{s}}_{o(kh) in}^t\ \left((kh) in=1,\dots, {linkin}_k\right) $$

where ${\mathbf{s}}_{o(kh) in}^t\in {R}^{L_{(kh) in}}$ represents slacks and is non-negative and *linkin*_*k*_ is the number of “as input” links from sub-division *k*. In the “as output” link value case, the linking activities are considered as an output from the preceding division, and shortages are accounted for in the output inefficiency:

3 $$ {\mathbf{z}}_{o(kh) out}^t={\mathbf{Z}}_{(kh) out}^t{\boldsymbol{\uplambda}}_k^t-{\mathbf{s}}_{o(kh) out}^t\ \left((kh) out=1,\dots, {linkout}_k\right) $$

where ${\mathbf{s}}_{o(kh) out}^t\in {R}^{L_{(kh) out}}$ represents slacks and is non-negative and *linkout*_*k*_ is the number of “as output” links from a sub-division *k*. Subsequently, the carry-over variables may be classified into four categories, with a detailed description in Table 1:

$$ {z}_{ok_l good}^{\left(t,t+1\right)}=\sum \limits_{j=1}^n{z}_{jk_l good}^{\left(t,t+1\right)}{\lambda}_{jk}^t-{s}_{ok_l good}^{\left(t,t+1\right)}\ \left({k}_l=1,\dots, {ngood}_k;\forall k,\forall t\right) $$

$$ {z}_{ok_l bad}^{\left(t,t+1\right)}=\sum \limits_{j=1}^n{z}_{jk_l bad}^{\left(t,t+1\right)}{\lambda}_{jk}^t+{s}_{ok_l bad}^{\left(t,t+1\right)}\ \left({k}_l=1,\dots, {nbad}_k;\forall k,\forall t\right) $$

$$ {z}_{ok_l free}^{\left(t,t+1\right)}=\sum \limits_{j=1}^n{z}_{jk_l free}^{\left(t,t+1\right)}{\lambda}_{jk}^t+{s}_{ok_l free}^{\left(t,t+1\right)}\ \left({k}_l=1,\dots, {nfree}_k;\forall k,\forall t\right) $$

$$ {z}_{ok_l fix}^{\left(t,t+1\right)}=\sum \limits_{j=1}^n{z}_{jk_l fix}^{\left(t,t+1\right)}{\lambda}_{jk}^t\ \left({k}_l=1,\dots, {nfix}_k;\forall k,\forall t\right) $$

4 $$ {s}_{ok_l good}^{\left(t,t+1\right)}\ge 0,{s}_{ok_l bad}^{\left(t,t+1\right)}\ge 0,\mathrm{a}\ {s}_{ok_l free}^{\left(t,t+1\right)}: free\forall {k}_l,\forall t $$

As already mentioned, the dynamic network DEA analysis considers the internal heterogeneity of DMUs, where the individual divisions are interconnected. Thus, the model may be used to assess overall efficiency, period efficiency, track changes in overall efficiency across periods, and divisional efficiency to track changes in efficiency across divisions between selected periods.

The objective function for the overall efficiency in the input-oriented model can be expressed by the following program [32]:

5 $$ {\theta}_o^{\ast }=\min\ \sum \limits_{t=1}^T{W}^t\left[\sum \limits_{k=1}^K{w}^k\left[1-\frac{1}{m_k+ lin\mathrm{k}{in}_k+{nbad}_k}\left(\sum \limits_{i=1}^{m_k}\frac{s_{iok}^{t-}}{x_{iok}^t}+\sum \limits_{(kh)_l=1}^{linkin_k}\frac{s_{o{(kh)}_l in}^t}{z_{o{(kh)}_l in}^t}+\sum \limits_{k_l=1}^{nbad_k}\frac{s_{o{k}_l bad}^{\left(t,t+1\right)}}{z_{o{k}_l bad}^{\left(t,t+1\right)}}\right)\right]\right] $$

where, *W*^*t*^(∀*t*) represents the weight to the period *t* and *w*^*k*^(∀*k*) represents the weight to the sub-division *k*. These weights satisfy the condition: $\sum \limits_{t=1}^T{W}^t=1$, $\sum \limits_{k=1}^K{w}^k=1,$ *W*^*t*^ ≥ 0 (∀*t*), *w*^*k*^ ≥ 0 (∀*k*). They are supplied exogenously.

The following program defines period efficiency in the input-oriented model:

6 $$ {\tau}_o^{t\ast }=\sum \limits_{k=1}^K{w}^k\left[1-\frac{1}{m_k+{linkin}_k+{nbad}_k}\left(\sum \limits_{i=1}^{m_k}\frac{s_{iok}^{t-}}{x_{iok}^t}+\sum \limits_{(kh)_l=1}^{linkin_k}\frac{s_{o{(kh)}_l in}^t}{z_{o{(kh)}_l in}^t}+\sum \limits_{k_l=1}^{nbad_k}\frac{s_{o{k}_l bad}^{\left(t,t+1\right)}}{z_{o{k}_l bad}^{\left(t,t+1\right)}}\right)\right]\left(\forall t\right) $$

The following program defines divisional efficiency in the input-oriented model:

7 $$ {\delta}_{ok}^{\ast }=\sum \limits_{t=1}^T{W}^t\left[1-\frac{1}{m_k+{linkin}_k+{nbad}_k}\left(\sum \limits_{i=1}^{m_k}\frac{s_{iok}^{t-}}{x_{iok}^t}+\sum \limits_{(kh)_l=1}^{linkin_k}\frac{s_{o{(kh)}_l in}^t}{z_{o{(kh)}_l in}^t}+\sum \limits_{k_l=1}^{nbad_k}\frac{s_{o{k}_l bad}^{\left(t,t+1\right)}}{z_{o{k}_l bad}^{\left(t,t+1\right)}}\right)\right]\ \left(\forall k\right) $$

Finally, period-divisional efficiency in the input-oriented model can be expressed by the following program:

8 $$ {\rho}_{ok}^{t\ast }=1-\frac{1}{m_k+{linkin}_k+{nbad}_k}\left(\sum \limits_{i=1}^{m_k}\frac{s_{iok}^{t-}}{x_{iok}^t}+\sum \limits_{(kh)_l=1}^{linkin_k}\frac{s_{o{(kh)}_l in}^t}{z_{o{(kh)}_l in}^t}+\sum \limits_{k_l=1}^{nbad_k}\frac{s_{o{k}_l bad}^{\left(t,t+1\right)}}{z_{o{k}_l\mathrm{b} ad}^{\left(t,t+1\right)}}\right)\ \left(\forall k,\forall t\right) $$

Further support for the output-oriented model is given by Kawaguchi et al. [33] and Ozcan & Khushalani [32], where detailed formulas are applied.

**Fig. 2:**
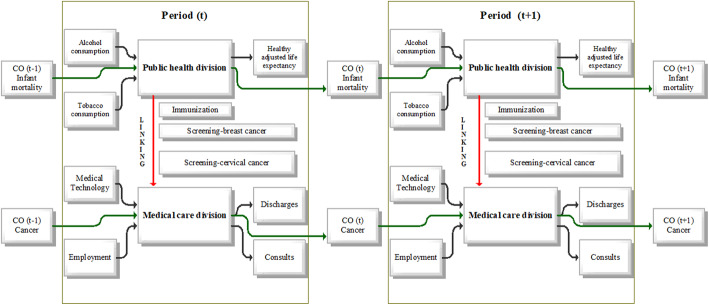
DNDEA model. Source: Prepared by authors based on Ozcan [5] and Ozcan & Khushalani [32]

The definitions and descriptive statistics of the inputs and outputs used in the analysis, are provided in Table 2. The DNDEA model being used in this paper, as shown in Fig. 2 considers a selected OECD country’s health system as DMU. This health system is conceived as having two sub-divisions – public health and medical care. Both sub-divisions have the same weights, and both are important parts of the health system [31].

**Table 2:** Classification of the indicators used and their descriptive statistical characteristics

		2000	2008	2016
Indicator	Definition	Avg / Min / Max	Avg / Min / Max	Avg / Min / Max
***Public health sub-division***				
**Inputs**				
Alcohol consumption	Annual alcohol consumption in litres per capita aged 15 years and older	9.5 / 1.5 / 14.2	9.7 / 1.5 / 14.2	8.9 / 1.3 / 13.2
Tobacco consumption	Annual consumption in grams of tobacco products (e.g. cigarettes, cigars, ...) per capita aged 15 years and over	26.1 / 12.4 / 35.0	22.1 / 10.8 / 39.7	18.6 / 7.6 / 27.3
**Output**				
Health-adjusted life expectancy	The average number of years that a person is expected to live in good health by taking into account years lived in less than full health due to disease and/or injury	68.0 / 60.6 / 72.5	70.1 / 64.4 / 73.8	71.1 / 66.0 / 74.8
**Links to medical care sub-division**				
Immunisation	Percentage of children under 1 year of age who were vaccinated against diphtheria, tetanus and whooping cough in that year	92.9 / 78.0 / 99.0	95.2 / 80.0 / 99.0	95.4 / 87.0 / 99.0
Breast cancer screening	Number of women aged 50–69 years who have undergone mammography in the last 2 years / number of women aged 50–69 years who answered survey questions	51.7 / 0.9 / 88.1	57.6 / 9.6 / 89.7	60.2 / 18.1 / 90.4
Cervical cancer screening	Number of women aged 20–69 years examined in the last 3 years / number of women aged 20–69 years who answered survey questions	56.1 / 6.3 / 90.6	59.9 / 14.9 / 85.9	61.9 / 18.2 / 86.6
**Carry-over**				
Infant mortality	Number of deaths of children under 1 year of age, calculated per 1000 births	6.8 / 3.0 / 28.4	4.6 / 1.8 / 15.7	3.9 / 0.7 / 12.1
***Medical care sub-division***				
**Inputs**				
Medical technology	Number of computer tomography devices per 1000,000 inhabitants	16.1 / 2.5 / 92.6	21.2 / 4.0 / 97.0	25.9 / 6.1 / 107.2
Employment in healthcare	Total number of doctors and nurses in health care calculated per 1000 inhabitants	10.0 / 2.3 / 16.7	11.3 / 2.7 / 18.5	12.5 / 3.8 / 22.0
**Outputs**				
Inpatient discharges	The average number of hospital discharges per 100,000 population for all diagnostic diseases	15,982.3 / 4016.6 / 25,933.3	16,105.1 / 4603.5 / 28,114.5	15,816.7 / 4617.2 / 25,685.9
Consultations	Average number of consultations per physician per capita	6.2 / 2.4 / 14.8	6.5 / 2.8 / 13.2	6.8 / 2.8 / 17.0
**Carry-over**				
Incidence of cancer	New cancer cases recalculated to 100,000 inhabitants	277.9 / 91.0 / 420.0	259.9 / 128.4 / 321.1	269.3 / 131.5 / 338.1

Source: Prepared by authors

The inputs to the public health sub-division include tobacco and alcohol consumption as the standard inputs used in previous studies e.g. [15, 17, 25], or [32]. The output for the public health sector is considered the health-adjusted life expectancy. Most studies in the field of measuring efficiency in health work with life expectancy (e.g. [1, 17, 18, 20, 32]). To overcome the shortcomings of previous studies outlined above, we propose using the health-adjusted life expectancy in the public health sub-division as the output. Using the HALE indicator better demonstrates and captures the quality of life in a country, as this indicator shows how many years a person is expected to live in good health [58–60]. Preventive services, such as immunisation, breast cancer screening, and cervical cancer screening, affect the quality of medical care services, but these are regulated at the level of the national healthcare division. Therefore we decide to use these variables not as inputs in medical care, but we use these preventive services as links between divisions. We can consider them as good links to the medical care area as they reduce the disease burden on the medical care area.

The input-oriented DNDEA model will be considered. It may minimise the inputs to achieve given results in the healthcare area, i.e. how a country should set its input indicators effectively to reach given outputs, namely the health-adjusted life expectancy. Since it is assumed that the adjustment of inputs cannot significantly increase this output at one time, but its level is given, the input-oriented models are used. There will be examined what should be the optimum level of public health input that corresponds to HALE. Similarly, there is involved a carry-over variable in the field of public health. In this case, such a variable is an indicator of infant mortality. The carry-over variable may affect the efficiency of a given division positively or negatively, but most likely not immediately in a given period. Its impact on the efficiency of public health will be reflected only after some time delay. The infant mortality indicator has an adverse effect on the level of healthcare in a given country, while rising values of this indicator have a negative impact on the overall efficiency of healthcare in a country.

The inputs to the medical care area include the number of persons working in the health care sector and the number of computer tomography devices in the country. These inputs represent capital, technology and labour and are commonly used in efficiency evaluation (e.g. [11, 15–18, 20, 28, 32–38, 41, 42]). Outputs from the medical care area represent the number of hospital discharges and consultations in a year. These are commonly used outputs in the evaluation efficiency of the medical care system (e.g. [2, 5, 9, 32, 35, 36], or [41, 42]). One of the advantages of the DNDEA model, which can be considered a contribution of the paper, is that we can consider so-called carry-over activities affecting the efficiency with time shift. In the medical care area, we include as carry-over the number of new cancer cases. As cancer is considered one of the most serious diseases of the last decade, which significantly burdens the health system, we decided to choose it for our analysis. The increasing number of new cancer cases represents an increased need to purchase new equipment and the increased number of medical staff in the future. Therefore, we consider it as undesirable (bad) carry-over of medical care area from one period to another.

The health-adjusted life expectancy (HALE) was collected from the Global Health Observatory database, which is World Health Organization’s data repository for health-related statistics for all member states. The HALE can be found under the “Indicator Groups” section in the sub-section “Healthy life expectancy”. All other indicators were collected from the online statistical platform of the OECD’s statistical databases. The Alcohol consumption and Tobacco consumption are available in the section “Health” and in the sub-section “Non-Medical Determinants of Health”. Immunisation, Breast cancer screening and Cervical cancer screening are available in the section “Health” in the sub-section “Health Care Utilisation” in the dimensions “Immunisation” or “Screening”, respectively. Infant mortality and Incidence of cancer are available in the section “Health” in the sub-section “Health Status” in the dimensions “Maternal and infant mortality” or “Cancer”, respectively. Medical technology refers to the number of computer tomography devices per one million inhabitants, and it is available in the section “Health” in the sub-section “Health Care Resources”. Employment in healthcare is available in the section “Health” in “Health Care Resources” in the dimension “Total health and social employment”. Inpatient discharges are available in the section “Health” in the sub-section “Health Care Utilisation” in the dimension “Hospital discharges by diagnostic categories”. Consultations are available in the section “Health” in the sub-section “Health Care Utilisation” and refer to the average number of consultations per physician per capita. All data were collected for 2000, 2008 and 2016 (as the last available data).

## Results

DNDEA analysis was conducted using DEA-Solver-Pro Version 13.0. An input-oriented DNDEA model was performed. The first goal in the analytical part was to find a suitable way to adjust the inputs to achieve efficiency. Ozcan & Khushalani [32] present options for adjusting inputs and outputs in this model. The focus is put on adjusting and re-evaluating the inputs at each level of HALE. The models’ results were interpreted using this indicator because the efficiency results are similar to those obtained when using the life expectancy at birth. The results of the DNDEA models with the assumption of constant returns to scale and variable returns to scale in 2000, 2008 and 2016 in each sub-division are shown in Table 3 and Table 4.

**Table 3:** Efficiency results - DNDEA model with CRS in OECD countries

DMU	Overall efficiency score			The efficiency of public health sub-division			The efficiency of medical care sub-division		
	2000	2008	2016	2000	2008	2016	2000	2008	2016
**AUS**	0.715	0.663	0.5879	0.8259	0.7757	0.6444	0.6042	0.5503	0.5315
**AUT**	1	1	1	1	1	1	1	1	1
**BEL**	0.703	0.7534	0.6141	0.7447	0.7498	0.5583	0.6613	0.757	0.6698
**CAN**	0.7876	0.8468	0.7836	0.8099	0.7703	0.7005	0.7653	0.9234	0.8667
**CZE**	1	0.9233	0.8965	1	0.8465	0.7931	1	1	1
**DNK**	0.6198	0.6214	0.6286	0.5818	0.642	0.6503	0.6577	0.6008	0.6068
**EST**	0.9362	0.7669	0.7527	0.8723	0.6247	0.65	1	0.909	0.8554
**FIN**	1	1	1	1	1	1	1	1	1
**FRA**	1	0.835	0.7032	1	0.6868	0.4872	1	0.9833	0.9191
**DEU**	0.6913	0.7918	0.7076	0.6175	0.8073	0.4996	0.765	0.7763	0.9157
**GRC**	0.9188	1	1	0.8376	1	1	1	1	1
**HUN**	0.8504	0.8307	0.9111	0.7008	0.6615	0.8223	1	1	1
**CHL**	1	1	1	1	1	1	1	1	1
**ISL**	0.8052	0.7758	0.7874	1	1	1	0.6105	0.5516	0.5748
**IRL**	0.6403	0.6801	0.6788	0.5994	0.661	0.6131	0.6813	0.6992	0.7444
**ISR**	0.9547	1	1	1	1	1	0.9094	1	1
**ITA**	0.7335	0.7838	0.6763	0.8119	0.8784	0.6671	0.655	0.6893	0.6855
**JPN**	1	1	1	1	1	1	1	1	1
**LVA**	1	0.8492	0.7935	1	0.7512	0.7351	1	0.9471	0.8519
**LTU**	1	1	0.8573	1	1	0.7146	1	1	1
**LUX**	0.8147	0.86	1	1	1	1	0.6294	0.7201	1
**MEX**	1	1	1	1	1	1	1	1	1
**NLD**	0.8111	0.884	1	0.8022	0.8304	1	0.82	0.9376	1
**NZL**	0.6679	0.7532	0.7144	0.6141	0.7252	0.6669	0.7217	0.7811	0.7619
**NOR**	0.9063	0.8456	0.8333	1	0.9284	1	0.8126	0.7629	0.6666
**POL**	0.9209	0.8849	0.7754	0.8419	0.7698	0.5508	1	1	1
**PRT**	0.9943	1	0.8877	0.9887	1	0.7755	1	1	1
**KOR**	1	1	0.8715	1	1	0.7429	1	1	1
**SVK**	1	1	0.8529	1	1	0.7071	1	1	0.9987
**SVN**	0.8784	1	1	0.8245	1	1	0.9322	1	1
**ESP**	0.8546	0.8538	0.8735	0.7091	0.7075	0.7469	1	1	1
**SWE**	0.958	1	1	1	1	1	0.916	1	1
**CHE**	0.6359	0.6766	0.5715	0.6786	0.7321	0.5512	0.5933	0.621	0.5917
**TUR**	1	1	1	1	1	1	1	1	1
**GBR**	0.9747	0.9274	0.8528	0.9493	0.8547	0.7056	1	1	1
**USA**	0.911	0.8995	0.8872	1	1	1	0.8221	0.7989	0.7744
**Average**	0.8801	0.8807	0.8472	0.8836	0.8723	0.8051	0.8766	0.8891	0.8893

Source: Prepared by authors

**Table 4:** Efficiency results - DNDEA model with VRS in OECD countries

DMU	Overall efficiency score			The efficiency of public health sub-division			The efficiency of medical care sub-division		
	2000	20,008	2016	2000	2008	2016	2000	2008	2016
**AUS**	0.7178	0.6642	0.6429	0.8427	0.7562	0.7327	0.5929	0.5721	0.5531
**AUT**	1	1	1	1	1	1	1	1	1
**BEL**	0.7453	0.9301	0.7541	0.7673	1	0.788	0.7234	0.8601	0.7203
**CAN**	1	1	1	1	1	1	1	1	1
**CZE**	1	1	0.9002	1	1	0.8003	1	1	1
**DNK**	0.6655	0.6295	0.6551	0.6161	0.6604	0.6839	0.7148	0.5986	0.6262
**EST**	0.9373	0.7745	0.8453	0.8745	0.6328	0.794	1	0.9162	0.8965
**FIN**	1	1	1	1	1	1	1	1	1
**FRA**	1	1	1	1	1	1	1	1	1
**DEU**	0.7355	1	1	0.7142	1	1	0.7569	1	1
**GRC**	0.9229	1	1	0.8459	1	1	1	1	1
**HUN**	1	1	1	1	1	1	1	1	1
**CHL**	1	1	1	1	1	1	1	1	1
**ISL**	0.867	0.7916	0.8221	1	1	1	0.734	0.5831	0.6442
**IRL**	0.7344	0.8987	0.8783	0.6211	0.7974	0.7566	0.8476	1	1
**ISR**	1	1	1	1	1	1	1	1	1
**ITA**	0.7372	0.8175	0.7339	0.8111	0.9381	0.7523	0.6632	0.697	0.7155
**JPN**	1	1	1	1	1	1	1	1	1
**LVA**	1	1	1	1	1	1	1	1	1
**LTU**	1	1	0.8578	1	1	0.7156	1	1	1
**LUX**	1	1	1	1	1	1	1	1	1
**MEX**	1	1	1	1	1	1	1	1	1
**NLD**	1	1	1	1	1	1	1	1	1
**NZL**	0.697	0.7754	0.7781	0.6726	0.768	0.7527	0.7215	0.7829	0.8035
**NOR**	0.9071	0.9328	0.8682	1	1	1	0.8142	0.8655	0.7364
**POL**	1	1	1	1	1	1	1	1	1
**PRT**	1	1	1	1	1	1	1	1	1
**KOR**	1	1	1	1	1	1	1	1	1
**SVK**	1	1	1	1	1	1	1	1	1
**SVN**	0.8853	1	1	0.8371	1	1	0.9335	1	1
**ESP**	1	1	1	1	1	1	1	1	1
**SWE**	1	1	1	1	1	1	1	1	1
**CHE**	0.9985	0.9999	0.9999	1	1	1	0.997	0.9999	0.9998
**TUR**	1	1	1	1	1	1	1	1	1
**GBR**	1	1	1	1	1	0.9999	1	1	1
**USA**	1	1	1	1	1	1	1	1	1
**Average**	0.9320	0.9504	0.9371	0.9334	0.9598	0.9382	0.9305	0.9410	0.9360

Source: Prepared by authors

The analysis results for the public health sub-division, medical care sub-division and overall health system for OECD countries under the assumption of CRS in Table 3 indicate that the average overall efficiency was 0.8801 in 2000. Twenty-one countries have achieved a higher efficiency compared to the average in 2000. On the other hand, fifteen countries have achieved a lower efficiency compared to the average in 2000. In 2008 the average overall efficiency was 0.8807, where nineteens countries have achieved a higher efficiency than the average in 2008, and seventeen countries have achieved a lower efficiency than the average in 2008. In 2016 the average overall efficiency was 0.8472, where twenty-one countries have achieved a higher efficiency than the average in 2016, and fifteen countries have achieved a lower efficiency than the average in 2016. According to the achieved score, we can mark as efficient in all three years: Austria (AUT), Finland (FIN), Chile (CHL), Japan (JPN), Mexico (MEX), and Turkey (TUR). According to the literature, the high-efficiency scores in these countries could be explained by healthcare reforms taken during the years 2000–2012 [33]. When we look at the efficiency scores of sub-divisions, we can see that the average efficiency of the public health sub-division was 0.8836 in 2000, 0.8723 in 2008 and 0.8051 in 2016. In 2016 sixteen countries achieved a higher efficiency compared to the average in the public health sub-division. In the medical care sub-division, the average was 0.8766 in 2000, 0.8891 in 2008 and 0.8893 in 2016. When we compare the level of public health sub-division and medical care sub-division efficiency score, we can see that the efficiency of the public health sub-division in 2016 was higher than medical care sub-division only in Australia (AUS), Denmark (DNK), Iceland (ISL), Norway (NOR) and United States (USA). In the case of Belgium (BEL), Canada (CAN), Czech Republic (CZE), Estonia (EST), France (FRA), Germany (DUE), Hungary (HUN), Ireland (IRL), Italy (ITA), Latvia (LVA), Lithuania (LTU), New Zealand (NZL), Poland (POL), Portugal (PRT), Korea (KOR), Slovak Republic (SVK), Spain (ESP), Switzerland (CHE), and United Kingdom (GBR), the medical care sub-division was more efficient. Comparing the results, we can say that the countries tend to be more efficient within the medical care sub-division. Both divisions were efficient in the case of Austria (AUT), Finland (FIN), Greece (GRC), Chile (CHL), Israel (ISR), Japan (JPN), Luxembourg (LUX), Mexico (MEX), Netherland (NDL), Slovenia (SVN), Sweden (SWE), and Turkey (TUR).

The results of the analysis for the public health sub-division, medical care sub-division and overall health system for OECD countries under the assumption of variable returns to scale in Table 4 indicate that the average overall efficiency was 0.9320 in 2000 and eleven countries have achieved a lower efficiency compared to the average in 2000. In 2008 the average overall efficiency was 0.9504, where nine countries have achieved a lower efficiency compared to the average in 2008. In 2016 the average overall efficiency was 0.9371, where eleven countries have achieved a lower efficiency compared to the average in 2016. According to the achieved score, we can mark as inefficient in all three years: Australia (AUS), Belgium (BEL), Denmark (DNK), Estonia (EST), Iceland (ISL), Ireland (IRL), Italy (ITA), New Zealand (NZL), Norway (NOR) and Switzerland (CHE). When we look at the efficiencies of sub-divisions, we can see that the average efficiency of the public health sub-division was 0.9334 in 2000, 0.9598 in 2008 and 0.9382 in 2016. In the medical care sub-division, the average was 0.9305 in 2000, 0.9410 in 2008 and 0.9360 in 2016. When we compare the level of public health sub-division and medical care sub-division efficiency score, we can see that the efficiency of the public health sub-division in 2016 was higher than medical care sub-division only in Australia (AUS), Belgium (BEL), Denmark (DNK), Iceland (ISL), Italy (ITA), Norway (NOR) and Switzerland (CHE). In the case of the Czech Republic (CZE), Estonia (EST), Ireland (IRL), Lithuania (LTU), New Zealand (NZL), and United Kingdom (GBR), the medical care sub-division was more efficient. In other countries, both divisions were efficient in 2016.

A closer examination of the results reveals some interesting findings of countries differences. In Table 5, we analyse Austria, Finland, Greece, Chile, Japan, Mexico and Turkey. These countries have reached higher than average efficiency scores in all three years examined. By further researching, we have tried to find out what these countries can have in common. These countries experienced several reforms in the reporting period, which had a significant impact on health care and the health system. Table 5 shows the level of individual indicators used in our models, health expenditures in US$ per capita and as % of GDP per capita all compared to the OECD average.

**Table 5:** Comparison of countries with above average efficiency (CRS model)

Indicator	Year	AUT	FIN	GRC	CHL	JPN	MEX	TUR	OECD Average
**Alcohol consumption**	**2000**	13.20	8.60	9.20	6.20	8.60	5.00	1.50	9.48
	**2008**	12.00	10.30	9.50	7.40	7.50	4.00	1.50	9.70
	**2016**	11.40	8.40	6.50	7.90	7.20	4.40	1.30	8.91
	**00–16**	−1.80	−0.20	−2.70	1.70	−1.40	−0.60	−0.20	− 0.57
**Tobacco consumption**	**2000**	24.30	23.40	35.00	33.00	27.00	12.40	32.10	25.74
	**2008**	23.20	20.40	39.70	29.80	21.80	10.80	27.40	22.18
	**2016**	24.30	15.00	27.30	24.50	18.30	7.60	26.50	18.83
	**00–16**	0.00	−8.40	−7.70	−8.50	−8.70	−4.80	−5.60	−6.92
**HALE**	**2000**	69.50	68.50	69.60	67.80	72.50	65.60	60.60	67.98
	**2008**	71.40	70.40	71.50	68.90	73.80	66.50	64.40	70.11
	**2016**	72.40	71.70	72.00	69.70	74.80	67.70	66.00	71.10
	**00–16**	2.90	3.20	2.40	1.90	2.30	2.10	5.40	3.12
**Immunisation**	**2000**	81.00	99.00	89.00	91.00	85.00	97.00	85.00	92.89
	**2008**	83.00	99.00	99.00	95.00	98.00	96.00	96.00	95.22
	**2016**	87.00	92.00	99.00	95.00	99.00	97.00	98.00	95.42
	**00–16**	6.00	−7.00	10.00	4.00	14.00	0.00	13.00	2.53
**Breast cancer screening**	**2000**	80.20	87.40	49.60	16.40	23.80	0.90	30.20	51.67
	**2008**	80.20	84.90	53.80	19.30	23.80	9.60	24.70	57.60
	**2016**	72.70	82.20	59.60	36.90	41.00	18.10	33.20	60.20
	**00–16**	−7.50	−5.20	10.00	20.50	17.20	17.20	3.00	8.53
**Screening cervical cancer**	**2000**	81.50	70.30	59.40	64.50	22.60	6.30	36.80	56.14
	**2008**	82.30	69.00	70.00	68.00	24.50	19.00	24.00	59.87
	**2016**	86.60	69.80	75.50	55.90	42.40	18.20	47.80	61.91
	**00–16**	5.10	−0.50	16.10	−8.60	19.80	11.90	11.00	5.77
**Infant mortality**	**2000**	4.80	3.80	5.90	8.90	3.20	20.80	28.40	6.77
	**2008**	3.70	2.60	2.70	7.80	2.60	15.10	15.70	4.63
	**2016**	3.10	1.90	4.20	6.90	2.00	12.10	10.00	3.87
	**00–16**	−1.70	−1.90	−1.70	−2.00	−1.20	−8.70	−18.40	−2.90
**Medical technology**	**2000**	26.09	13.52	25.48	10.20	92.62	2.45	4.89	16.11
	**2008**	29.68	16.45	31.05	10.20	96.97	4.01	10.68	21.21
	**2016**	29.07	24.20	36.66	14.76	107.17	6.12	14.53	25.94
	**00–16**	2.98	10.68	11.18	4.56	14.55	3.67	9.64	9.83
**Employment**	**2000**	11.01	13.21	7.12	2.34	10.36	3.78	2.36	10.01
	**2008**	12.13	15.86	9.39	2.70	11.73	4.24	3.00	11.30
	**2016**	13.12	17.62	9.84	5.02	13.77	5.25	3.76	12.49
	**00–16**	2.11	4.41	2.72	2.68	3.41	1.47	1.40	2.47
**Hospital discharges**	**2000**	25,933.30	21,444.70	16,242.50	10,057.90	10,326.50	4016.60	7711.90	15,982.29
	**2008**	28,114.50	18,405.90	20,050.20	9639.60	11,161.70	4603.50	13,629.90	16,105.10
	**2016**	25,310.00	16,555.40	19,645.60	9000.00	12,638.80	4617.20	16,785.70	15,816.70
	**00–16**	− 623.30	− 4889.30	3403.10	−1057.90	2312.30	600.60	9073.80	− 165.59
**Consultations**	**2000**	6.70	4.30	4.30	2.40	14.40	2.50	2.80	6.17
	**2008**	6.90	4.30	4.00	3.00	13.20	2.80	6.70	6.52
	**2016**	6.60	4.30	4.00	3.50	12.80	2.90	8.60	6.85
	**00–16**	−0.10	0.00	−0.30	1.10	−1.60	0.40	5.80	0.68
**Cancer**	**2000**	237.00	247.00	162.00	176.70	201.10	91.00	144.80	277.87
	**2008**	250.60	250.10	162.00	176.70	201.10	128.40	144.80	259.93
	**2016**	254.10	256.80	163.00	175.70	217.10	131.50	205.10	269.26
	**00–16**	17.10	9.80	1.00	−1.00	16.00	40.50	60.30	−8.61
Health expenditure *	**2000**	2703.628	1828.671	1413.592	667.5	1917.971	484.387	425.6	1760.69
	**2008**	4018.27	3231.159	2895.361	1113.972	2852.863	846.849	836.811	2962.61
	**2016**	5273.243	4117.913	2262.788	1892.592	4585.388	1020.301	1092.466	3867.04
	**00–16**	2569.62	2289.24	849.20	1225.09	2667.42	535.91	666.87	2106.35
Health expenditure % of GDP **	**2000**	9.20	6.84	7.24	7.04	7.15	4.45	4.62	7.16
	**2008**	9.73	8.08	9.38	6.74	8.20	5.70	5.26	8.16
	**2016**	10.44	9.49	8.45	8.19	10.84	5.47	4.31	8.88
	**00–16**	1.23	2.66	1.21	1.15	3.69	1.02	−0.31	1.72

* Curent expenditure on health, per capita, US$ purchasing power parities (current prices)

** Current expenditure on health, % of gross domestic product

Source: prepared by authors

Austria’s health system is based on social insurance scheme. The federal government, social health insurance funds, the states (Länder) and municipalities all are responsible to some extent for the organisation of public and medical care, and all contribute to the health budget framework [61]. The fragmentation of the organisation of Austria’s health system has been adjusted through the new “target-based governance” system started in 2013 within a view of further reforms in the pipeline. Among the countries with the highest efficiency scores, health spending was the highest and well above the OECD average. Austria spent 2703.63 US$ per capita in 2000, and health expenditures almost doubled to 5273.24 US$ per capita by 2016, representing 10.4% of GDP (8.9% OECD average). In 2016 HALE was 72.4 years, 2.9 years higher than in 2000 and 1.3 years above the OECD average. The gaps remain in vaccination coverage (immunisation level) compared to the OECD average during the years. As is stated by OECD [61], behavioural risk factors and the ageing population will be the challenge of Austria’s health system. Despite a reduction since 2000 the alcohol consumption remains above the OECD average. Tobacco consumption among 15-year-olds remains above the OECD average, and overweight, obesity, and unhealthy diets represent serious health concerns [62]. The fragmented structure of the health system generally tends to a good level of health, but it seems more costly compared to other OECD countries, which allows a more detailed examination of efficiency at the regional level.

Finland’s health system has implemented several reforms in recent years, further described by Keskimäki et al. [63]. Health spending is less than in Austria but still above the OECD average. The health system faces rapid technological change, an ageing population and behavioural health risks. Relatively high-efficiency scores are complemented by the low levels of health spending. In 2001, Finland’s government published a new health strategy, “Government Resolution on the Health 2015 public health programme” [64]. In 2016 HALE was 71.7 years, 3.2 years higher than in 2000, and 0.6 above the OECD average, reflecting the positive impacts of government policies and interventions of implemented reforms. The progress can also be seen in risky factors as alcohol consumption and tobacco consumption. Both indicators have reached levels below the OECD average during the years. As stated by OECD [65], obesity rates have shown modest results and can be considered as one of the main public health concerns in the coming years. Finland has reached significant improvement in infant mortality since 2000. Health expenditure has more than doubled since 2000, while the share of expenditure expressed as % of GDP has also increased by more than 2.6%, which can be assessed positively in the health system’s performance. OECD [65] refers to the large social inequalities in life expectancy not only by gender but also by socioeconomic status and the level of education.

Greece scored best among the analysed group of countries in the Malmquist results (see Table 7). Efficiency results also indicate a good level of health care and the health status of the population over the reporting period. We would like to point out that Greece has the biggest efficiency improvement between 2000 and 2016, that is, during the period when the country was the most adversely affected by the Great Recession of all OECD countries. Among the OECD countries, Greece’s health system in recent years has undergone a major transformation. Health reforms were linked to the economic crisis and subsequently to the series of Economic Adjustment Programmes since 2010. The huge step forward was establishing the National Organisation for the Provision of Health Services to foster unified health insurance [66]. The important role also plays a new health technology assessment agency. Greece has reached the highest wasteful spending on pharmaceuticals in 2009. The government has established legislation for more than two million people who lost health insurance during the previous economic crisis. During the period, a rapid decrease in health spending can be observed. The current levels of health expenditures stabilised just before 2016. In 2016, Greece spent 2262.79 US$ per capita, well below the OECD average, representing 8.5% of GDP (8.9% OECD average). Greece has relatively high and encouraging levels of immunisation, as well as HALE at 72 years in 2016, representing an improvement from 69.6 years in 2000. The policy agreements and financial support from the European Structural and Investment Funds and the European Regional Development Fund have moved the health system to a higher level. In the area of technological change, this is visible in the increase in the number of CT devices per one million inhabitants, which reached 36.7, well above the OECD average in 2016 (25.9). According to Economou et al. [67], alcohol consumption,tobacco consumption, and mainly socio-economic disproportional gaps, will play an important health risk factor and continue challenging Greece’s health system.

Over the past years, there were significant improvements in Chilean’s health system and public health architecture. To improves the Chileans’ health status government has introduced the framework of policies toward significant change. The National Health Fund – Fondo Nacinal de Salud, known as FONASA, is responsible for health coverage. In 2005, Chile implemented a guaranteed package with access to the treatments, known as AUGE (see Auraaen et al. [68]; Barasa et al. [69]). The improvement in the health system is also associated with the Ricardo Soto Law introduced in 2015, re-examined in 2017. Despite many successes due to reforms, the health system is still below indicators that are at least similar to the OECD average. Health expenditures in 2016 was 1892.59 US$, well below the OECD average, representing more than 8.1% of GDP. In 2016, infant mortality was relatively high compared to the OECD average. According to OECD [70], Chile faces a number of natural hazards, earthquakes, tsunamis, wildfires and landslides, which represent high public health risks. Tuxedo rates are still very high compared to the OECD average. Alcohol consumption is below the OECD average but rising since 2000. Based on OECD [70], Chilean’s health system recorded several significant improvements but addressing obesity and overweight need to be a priority in the coming future. More can also be done in terms of cancer screening. Chile has low screening rates for cervical and breast cancers compared to the OECD average.

A more detailed look at the individual indicators shows that Japan has the longest living and seems to have the healthiest population at all. Healthy adjusted life expectancy at birth was 74.8 years in 2016, 3.7 years higher than the OECD average. The alcohol and tobacco consumption rates are also below the OECD average [71]. Based on OECD [72] Japan’s obesity and the overweight rate are the lowest in the OECD. But on the other hand, the ageing population seems to be a challenge for Japan’s health system in the next years. Health Japan 21, focused on primary prevention [72], is the winner of success in the health sector. In 2003 legal act was implemented, started developing the strategy. Health Japan 21 strategy has already two terms (first started from 2000 to 2013, and second started from 2013 to 2022). The second term brings a new framework consisting of 53 targets. Health Japan 21 is considered to be a comprehensive framework implemented at the local level and aimed at promoting healthy lifestyles. Japan, also like Chile, faces a number of natural hazards as earthquakes, floods, and tsunamis, which represent high public health risks. The level of immunisation is very high, and the infant mortality rate is positively below the OECD average.

Investment in health care in Mexico has increased in recent years [73]. Health expenditure grew from 4.5% of GDP in 2000 to 5.5% of GDP in 2016. The fundamental part of the health system in Mexico is the cluster of subsystems [74]. Individuals affiliation is determined by their job. There are fragmented packages and different sets of providers belonging on the one hand to Seguro Popular or on the other to the Institute for Social Security and Services for State Employees [75]. As is stated by OECD [73], the health system should change the position narrowing to high-quality care provision. Mexico has reached the improvement in HALE, from 65.6 years in 2000 to 67.7 in 2016. Since 2000 the rapid decrease in infant mortality is evident but remains well above the OECD average. The immunisation level in Mexico is above the OECD average. The OECD [73] emphasise, in the face of health system challenges, several reforms to Mexico’s health system.

Several health reforms were implemented by the Turkish government in the last years [76]. Health Transformation Programme began in 2003 and was complemented by significant health investments. Since 2003 the access to health care has spread rapidly. Among other things, these reforms led to an increase in health expenditure from 2000 to 2016. In 2016, they were at 1092.47 US$, still well below the OECD average. Health expenditures in terms of % of GDP went from 4.6% in 2000 to 4.3% in 2016. HALE, Immunisation and Infant mortality rates have improved impressively since 2000. Despite the success, health indicators are still below the OECD average, and there is still room for improvement. The OECD [77] proposes to continue the set the trend and focus on ensuring the quality of health services.

On the other hand, between the countries with the lowest efficiency (Table 3), we can see Denmark in 2000. The reason was the lowest efficiency within the public health sub-division, where a relatively high level of alcohol and tobacco consumption could be seen compared to the level of HALE. According to the OECD [78] report, harmful alcohol use is associated with numerous adverse health outcomes, early retirement, and social consequences. It also contributes to premature death, morbidity and disability. But in the last years, the positive development in this area could be seen. Alcohol consumption in Denmark fell by 27% between 2000 and 2013, and this way, the efficiency slightly increased. The Danish government prepared national law and prohibited the sale of alcohol to anyone under the age of 16. The Danish Health Authority’s alcohol control activities focus mainly on preventing alcohol abuse in specific settings through several projects, provides evidence-based recommendations concerning alcohol prevention to municipalities.

Another country with the lowest efficiency is Australia in 2016, where the reason is the lowest efficiency within the medical care sub-division. As the source of inefficiency, we can consider the inefficient usage of technologies and employees compared to the outputs. Australia is a country with a well-developed system and medical infrastructure. But when we compare the level of discharges and consultations, we can see that other countries can provide a similar level of services with a lower level of technologies and employees. Therefore, in the case of this country, the space for increase of effective usage of technologies and employees could be seen. Another fact is that despite the high level of technology, the increasing tendency of new cancer cases could be seen. According to the report of OECD [79], it is necessary to implement a uniform electronic health record system to improve the transfer of information between health care services. It is also important to create a primary health care eco-system around general practitioners and promote their role as care coordinators for patients with chronic diseases. They also suggest providing financial incentives for doctors to provide integrated care, improve the quality and outcomes of health care, and engage more in preventive health care.

However, we consider it important to highlight that in our analysis, we did not only remain with the outcomes of the CRS or VRS models, but we carried out a deeper comparison of countries also in the context of the exclusion of outlier observations, which can be considered as different from previous studies. The sensitivity of the DEA analysis to individual data also confirms the importance of additional verification of the results, excluding outlier observations, since AUT, GRC, CHL, JPN, MEX and TUR were highly represented by outliers.

The DEA models are sensitive to extreme values (outliers) for inputs and outputs [54]. Therefore, an additional analysis by using box-plot charts was performed. The results of the DNDEA model with VRS are presented in Table 6, taking into account the outliers of individual inputs and outputs in the selected countries. Consequently, the number of OECD countries was reduced. To consider the deletion of the countries with extreme values, the sample was reduced to 23 OECD countries. The results show that the average efficiency increased by approximately 4% after the outliers were excluded. In all countries, an increase in efficiency after eliminating outliers may be observed, and in either case, there was no decrease in efficiency. The most significant efficiency growth was found in Australia (AUS), Italy (ITA) and New Zealand (NZL). It may be assumed that the consideration of countries with extremely low and/or extremely high values of inputs and outputs influenced the values of examined efficiency in these countries. As we mentioned above, after eliminating the extreme values of the individual variables, the average efficiency increased. It may also be affected by the fact that the sample size has decreased significantly after eliminating extreme values. Effectively this can be formally stated as follows that the extreme values may affect the level of health system efficiency score. The elimination process of outliers has led to higher homogeneity of the results, as evidenced by the lower standard deviation values of overall efficiency during the analysed period. For instance, in 2016, variability decreased from 0.1057 to 0.0738. These findings reinforce the general belief that the DEA models are sensitive to outliers. For these reasons, the analysis of outliers in DEA models may be desirable.

**Table 6:** Comparison of the overall efficiency results before and after considering the deletion of outliers

DMU	Overall efficiency score - all countries			Overall efficiency score-reduced sample			Total Efficiency Change		
	2000	2008	2016	2000	2008	2016	2000	2008	2016
**AUS**	0.7178	0.6642	0.6429	0.9998	0.8443	0.8627	39.29%	27.12%	34.19%
**AUT**	1	1	1						
**BEL**	0.7453	0.9301	0.7541	0.797	0.9334	0.8668	6.94%	0.35%	14.94%
**CAN**	1	1	1						
**CZE**	1	1	0.9002						
**DNK**	0.6655	0.6295	0.6551	0.71	0.6716	0.6776	6.69%	6.69%	3.43%
**EST**	0.9373	0.7745	0.8453	1	1	1	6.69%	29.12%	18.30%
**FIN**	1	1	1	1	1	1	0.00%	0.00%	0.00%
**FRA**	1	1	1	1	1	1	0.00%	0.00%	0.00%
**DEU**	0.7355	1	1						
**GRC**	0.9229	1	1						
**HUN**	1	1	1	1	1	1	0.00%	0.00%	0.00%
**CHL**	1	1	1						
**ISL**	0.867	0.7916	0.8221						
**IRL**	0.7344	0.8987	0.8783	0.8661	1	1	17.93%	11.27%	13.86%
**ISR**	1	1	1						
**ITA**	0.7372	0.8175	0.7339	1	1	1	35.65%	22.32%	36.26%
**JPN**	1	1	1						
**LVA**	1	1	1	1	1	1	0.00%	0.00%	0.00%
**LTU**	1	1	0.8578	1	1	1	0.00%	0.00%	16.58%
**LUX**	1	1	1	1	1	1	0.00%	0.00%	0.00%
**MEX**	1	1	1						
**NLD**	1	1	1	1	1	1	0.00%	0.00%	0.00%
**NZL**	0.697	0.7754	0.7781	0.9999	0.9999	0.9999	43.46%	28.95%	28.51%
**NOR**	0.9071	0.9328	0.8682	1	1	1	10.24%	7.20%	15.18%
**POL**	1	1	1	1	1	1	0.00%	0.00%	0.00%
**PRT**	1	1	1	1	1	1	0.00%	0.00%	0.00%
**KOR**	1	1	1						
**SVK**	1	1	1						
**SVN**	0.8853	1	1	0.9359	1	1	5.72%	0.00%	0.00%
**ESP**	1	1	1	1	1	1	0.00%	0.00%	0.00%
**SWE**	1	1	1	1	1	1	0.00%	0.00%	0.00%
**CHE**	0.9985	0.9999	0.9999	0.9998	1	1	0.13%	0.01%	0.01%
**TUR**	1	1	1						
**GBR**	1	1	1	1	1	1	0.00%	0.00%	0.00%
**USA**	1	1	1	1	1	1	0.00%	0.00%	0.00%
**Average**	0.932	0.9504	0.9371	0.9699	0.9761	0.9742	4.07%	2.70%	3.96%
**Standard deviation**	0.1106	0.0999	0.1057	0.0742	0.0732	0.0738			

Source: Prepared by authors

Using Malmquist Index (MI), we can further observe the changes in the efficiency among countries over time. The detailed specification of the calculation of MI is given in the study by Färe et al. [56]. The MI values may be greater than or less than 1. The MI higher than one indicates that the efficiency in the monitored DMU has improved over the reporting period. On the other hand, the MI lower than one indicates that the efficiency has worsened over time. The overall MI can be decomposed into two effects: The Frontier Shift (FS) effect and the Catch-up (CU) effect. The FS effect describes an improvement in efficiency due to the innovation, while the CU effect provides an improvement in efficiency due to improved operations and management of the healthcare system (or management of public health sub-division or medical care sub-division). Table 7 shows MI results with the assumption of a CRS. In a comprehensive statement, there was an average 5% improvement in OECD health performance between 2000 and 2016. The progress in the overall total factor productivity index was caused by the 16% growth in the relative technical efficiency (CU effect) and negative innovation (FS) effect by the 4%, which led to the shift of production possibility frontier. The overall progress was positively influenced by the progress of 19% in the case of the public health sub-division and by the deterioration of 8% in the medical sub-division. The progress in the MI index in the public health sub-division was caused by the progress of 26% in the relative technical efficiency and the negative innovation effect by 5%. In the case of the medical care sub-division, the deterioration was caused by the decline in the relative technical efficiency by 8% and the positive innovation effect by 1%. The frontier shift effect representing the impact of innovation was positive only within the medical care sub-division. We can suppose that thanks to innovation in information technologies, the medical care sub-division was able to offer more appropriate services, enabling them to take their services closer to patients and so increase their efficiency. Overall, 25 OECD countries achieved an improvement (MI > 1) in performance. On the other hand, only nine countries recorded a decline (MI < 1) over the reporting period: Lithuania, Slovakia, Chile, the United Kingdom, Estonia, Norway, Poland, Latvia and France. Israel and Belgium did not record any change in performance during the reporting period. The highest growth can be seen in the case of Greece, which achieved an efficiency improvement of 21% between the years 2000 and 2016. On the other hand, France achieved the highest decline of 10%. Future research could extend the results of this work towards a number of directions.

**Table 7:** The Malmquist Index levels for the DNDEA model with CRS

DMU	Overall			Public health			Medical care		
	MI	CU	FS	MI	CU	FS	MI	CU	FS
**GRC**	1.21	1.34	1.09	1.40	1.28	1.09	1.04	1.04	1
**NLD**	1.19	1.16	1.23	1.30	1.17	1.12	1.09	0.99	1.10
**LUX**	1.18	1.11	1.26	1.29	1.29	1	1.09	0.86	1.26
**KOR**	1.14	1.51	0.86	1.18	1.37	0.86	1.10	1.10	1
**TUR**	1.13	1.27	1.00	1.14	1.14	1	1.11	1.11	1
**NZL**	1.13	1.19	1.07	1.37	1.32	1.04	0.93	0.90	1.03
**SVN**	1.13	1.11	1.14	1.37	1.24	1.10	0.93	0.89	1.04
**CAN**	1.11	1.24	0.99	1.25	1.34	0.93	0.99	0.93	1.06
**CZE**	1.10	1.36	0.89	1.23	1.39	0.89	0.98	0.98	1
**AUS**	1.09	1.44	0.83	1.28	1.45	0.88	0.93	1.00	0.94
**HUN**	1.09	1.09	1.08	1.28	1.18	1.08	0.93	0.93	1
**ISL**	1.08	1.21	0.97	1.39	1.39	1	0.85	0.87	0.97
**DEU**	1.08	1.19	0.98	1.14	1.26	0.90	1.03	0.94	1.09
**USA**	1.08	1.20	0.97	1.24	1.24	1	0.94	0.97	0.97
**ESP**	1.07	1.12	1.03	1.25	1.21	1.03	0.92	0.92	1
**IRL**	1.07	1.08	1.06	1.26	1.24	1.01	0.91	0.87	1.05
**JPN**	1.06	1.13	1.00	1.27	1.27	1	0.89	0.89	1
**ITA**	1.05	1.20	0.93	1.16	1.28	0.91	0.96	0.94	1.02
**CHE**	1.04	1.21	0.90	1.15	1.27	0.90	0.95	0.95	1.00
**PRT**	1.04	1.23	0.89	1.21	1.36	0.89	0.90	0.90	1
**AUT**	1.04	1.08	1.00	1.17	1.17	1	0.93	0.93	1
**MEX**	1.02	1.04	1.00	1.16	1.16	1	0.90	0.90	1
**SWE**	1.01	0.98	1.04	1.11	1.11	1	0.92	0.88	1.04
**FIN**	1.01	1.02	1.00	1.25	1.25	1	0.81	0.81	1
**DNK**	1.01	1.00	1.02	1.26	1.19	1.06	0.81	0.84	0.96
**ISR**	1.00	0.96	1.05	1.13	1.13	1	0.89	0.84	1.05
**BEL**	1.00	1.14	0.87	1.11	1.29	0.87	0.89	0.89	1.01
**LTU**	0.99	1.17	0.85	1.13	1.33	0.85	0.88	0.88	1
**SVK**	0.99	1.16	0.84	1.04	1.23	0.84	0.94	0.94	1.00
**CHL**	0.99	0.97	1.00	1.07	1.07	1	0.91	0.91	1
**GBR**	0.98	1.12	0.86	1.08	1.26	0.86	0.89	0.89	1
**EST**	0.98	1.20	0.80	1.20	1.39	0.86	0.80	0.87	0.92
**NOR**	0.97	1.04	0.91	1.14	1.14	1	0.83	0.92	0.91
**POL**	0.97	1.15	0.81	1.04	1.29	0.81	0.90	0.90	1
**LVA**	0.96	1.16	0.79	1.12	1.31	0.86	0.82	0.88	0.92
**FRA**	0.90	1.22	0.67	1.01	1.44	0.70	0.81	0.84	0.96
**Geometric mean**	1.05	1.16	0.96	1.19	1.26	0.95	0.92	0.92	1.01

Source: Prepared by authors

**Fig. 3:**
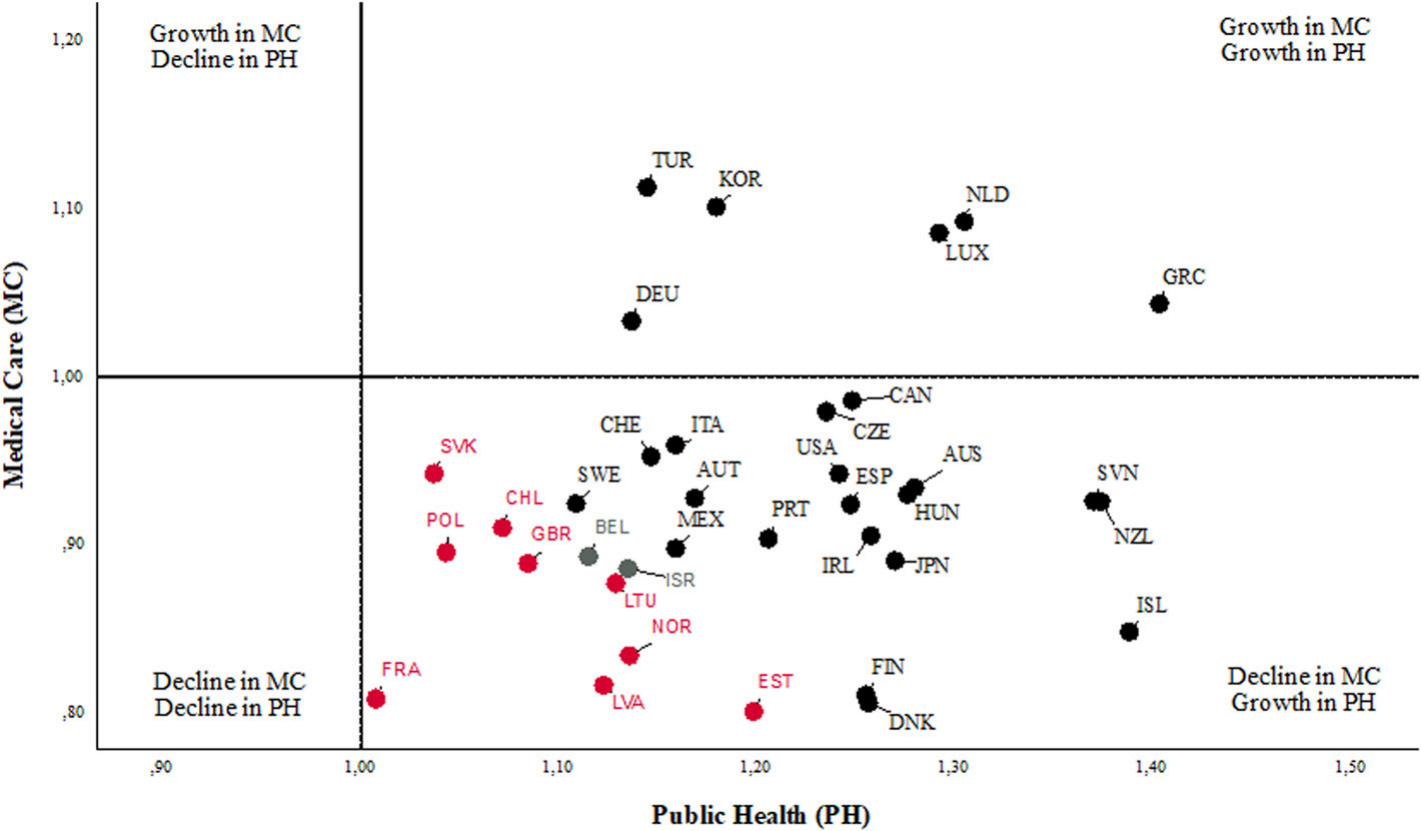
The MI results at CRS. Note: Red indicates those countries that have achieved an overall decline in health performance over the reporting period (i.e. MI < 1, regress). Grey indicates countries where there has been no overall change in health performance. Black indicates countries with overall progress in healthcare performance (i.e. MI > 1, progress). Source: Prepared by authors

**Fig. 4:**
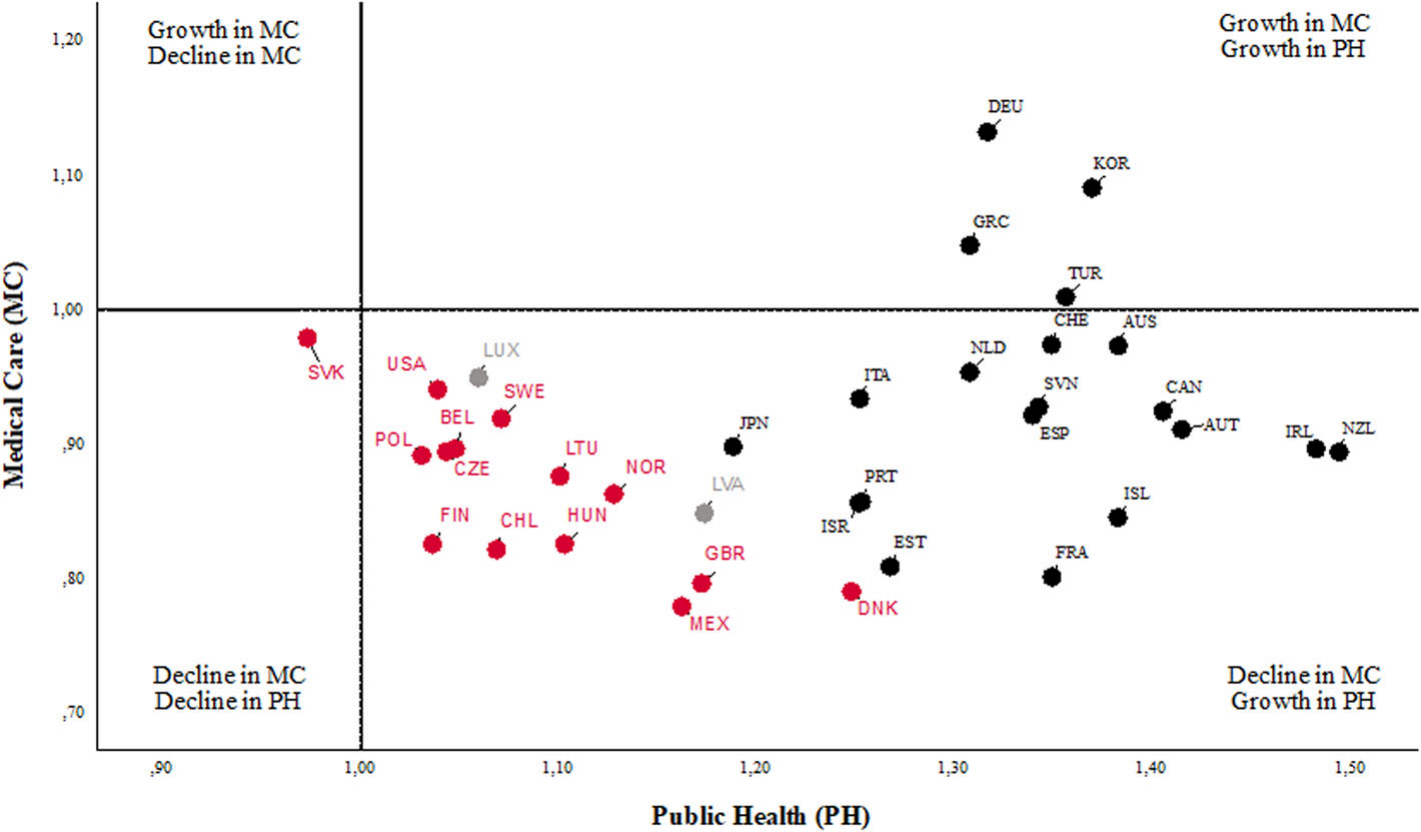
The MI results at VRS. Note: Red indicates those countries that have achieved an overall decline in health performance over the reporting period (i.e. MI < 1, regress). Grey indicates countries where there has been no overall change in health performance. Black indicates countries with overall progress in healthcare performance (i.e. MI > 1, progress). Source: Prepared by authors

In a comprehensive assessment of OECD countries, an improvement in the performance of 19% in the public health sub-division, with a decrease in performance of 8% in the medical care sub-division, is evident. Figure 3 and Fig. 4 show a comparison of the Malmquist Index levels within the public health and medical care sub-divisions of OECD countries between 2000 and 2016 for the DNDEA model with the assumption of CRS and VRS, respectively.

The figures are divided into four quadrants based on growth or a decline in performance in the public health sub-division and medical care sub-division. The country classification of the DNDEA model with CRS narrows to two areas - the second and fourth quadrants. Turkey, Korea, Germany, Luxembourg, the Netherlands and Greece saw performance growth in both sub-divisions. At the same time, these countries also saw an overall increase in performance over the reporting period. Belgium and Israel saw no increase or decrease in overall performance over the period under review. These countries are part of the fourth quadrant. In these countries, there has been a decline in performance in the medical care sub-division and an increase in performance in the public health sub-division.

Slovakia, Poland, Chile, the United Kingdom, Lithuania, Norway, Latvia, Estonia and France experienced an overall decline in performance over the reporting period, mainly related to a decline in the medical care sub-division. Interestingly, there was no decline in performance in the public health sub-division in any country over the reporting period.

Table 8 shows the classification of countries based on the MI and its components for CRS and VRS, which show that in the case of the DNDEA model, the CRS progression in performance in all countries (except Sweden and Denmark) results from increased efficiency levels.

**Table 8:** Profile for OECD countries based on the Malmquist Index values and its components

	EC and TC regress	EC regress and TC progress	EC progress and TC regress	EC and TC progress
**DNDEA_HALE_CRS model**				
Productivity progress M > 1	–	SWE, DNK	KOR, CAN, CZE, AUS, ISL, DEU, USA, ITA, CHE, PRT, FIN, TUR, JPN, AUT, MEX	GRC, NLD, LUX, NZL, SVN, HUN, ESP, IRL
Productivity regress M < 1	CHL	ISR	LTU, SVK, GBR, EST, NOR, POL, LVA, FRA, BEL	–
**DNDEA_HALE_VRS model**				
Productivity progress M > 1	–	–	KOR, TUR, AUS, CAN, AUT, NLD, ESP, ISL, FRA, PRT, ISR, JPN, EST, LUX	DEU, GRC, NZL, IRL, CHE, SVN, ITA
Productivity regress M < 1	LVA, DNK, SWE, USA, SVK, GBR, POL, HUN, MEX, CHL, FIN	BEL	NOR, LTU, CZE	–

Source: Prepared by authors

In view of all that has been mentioned so far, the decomposition of the Malmquist Index allows us to explore two areas: efficiency change (CU effect) and technology change (FS effect) for each OECD country. Changes in the CU effect point to an increase in performance in this area of 16% overall for all OECD countries within the reporting period, with the reported result indicating a shift of these countries to the efficiency frontier. Conversely, the average change in FS effect recorded a decline by 4% for OECD countries implying that the efficiency frontier is shifting negatively, which may cause further technological decline (regress). These results agree well with existing studies, by Dimas et al. [38] and Mitropoulos [41], on the results suggesting progress in productivity in OECD countries, which is related to the change in efficiency than the change in technologies.

## Discussion

The main objective of this study was to quantify and compare the efficiency scores of OECD health systems during 2000, 2008 and 2016 by applying the Dynamic Network Data Envelopment Analysis in the sub-divisions of public health and medical care provision. Consequently, an examination of the efficiency of health systems at macroeconomic and microeconomic levels, as well as investigating the causal links between them and their impact on overall efficiency, had been explored. This research trajectory also benefits methodologically. It is hoped that this study will lead to new insights into the elimination of one of the disadvantages of traditional DEA analyses - sensitivity to input and output variables’ selection (and outliers), a number of inputs and outputs, measurement errors (for more details in the studies of, e.g. [34–36, 42], or [80]), as well as on measurement variations [37].

Healthcare is a complex heterogeneous system influenced both by political decisions within the country and by the health processes in different types of healthcare facilities. Their quality and efficiency are also influenced by the type of hospital ownership, financial flows linked to therapeutic and diagnostic processes, geographical and socio-economic determinants, population structure, the political situation in the country, etc. Thus, investigating efficiency in separate sub-divisions has enabled us to identify the critical points of efficiency and create space for the more efficient redistribution of available resources in the health system than assessing the quality of healthcare provided by patients. The input-oriented DNDEA model was chosen to achieve the goal of this study. This model was also applied by Färe et al. [55]. Our research is the first step towards a more profound understanding of DNDEA analysis in the health system consisting of two main sub-divisions: public health and medical care sub-division, considering health-adjusted life expectancy as the main output of public health sub-division. The significance of HALE is described by several authors in their studies (e.g. [58–60]). Although there were some limitations in the form of missing data, to solve this problem, Anderson et al. [27] recommend using data closest to the reference year or the period for which the analysis is realised. This issue should be anticipated and addressed in future studies.

The analyses’ results show many interesting findings that may be interpreted both in a comprehensive evaluation of the efficiency of health systems and in the individual divisions. Subsequently, they may compare the macroeconomic and microeconomic spheres in this system. The main achievements, including contributions, may be summarised as follows.

The analysis results for the public health sub-division, medical care sub-division and overall health system for OECD countries under the assumption of CRS indicate that the average overall efficiency was 0.8801 in 2000, 0.8807 in 2008 and 0.8472 in 2016. According to the achieved score, we can mark as efficient in all three years: Austria, Finland, Chile, Japan, Mexico, and Turkey. The favourable development in these countries could be influenced by healthcare reforms taken in specified countries during the years 2000–2012. When we look at the efficiencies of sub-divisions, we can see that the average efficiency of the public health sub-division was 0.8836 in 2000, 0.8723 in 2008 and 0.8051 in 2016. In the medical care sub-division, the average was 0.8766 in 2000, 0.8891 in 2008 and 0.8893 in 2016. Comparing the results, we can say that the countries tend to be more efficient within the medical care sub-division.

The analysis results under the assumption of VRS indicate that the average overall efficiency was 0.9320 in 2000, 0.9504 in 2008 and 0.9371 in 2016. According to the achieved score, we can mark as inefficient in all three years: Australia, Belgium, Denmark, Estonia, Israel, Ireland, Italy, New Zealand, Norway and Switzerland. When we look at the efficiencies of sub-divisions, we can see that the average efficiency of the public health sub-division was 0.9334 in 2000, 0.9598 in 2008 and 0.9382 in 2016. In the medical care sub-division, the average was 0.9305 in 2000, 0.9410 in 2008 and 0.9360 in 2016. When we compare the level of public health sub-division and medical care sub-division efficiency score, we can see that the efficiency of the public health sub-division in 2016 was higher than medical care sub-division only in Australia, Belgium, Denmark, Iceland, Italy, Norway and Switzerland. The medical care sub-division was more efficient than the public health sub-division in the Czech Republic, Estonia, Ireland, Lithuania, New Zealand, and the United Kingdom. In other countries, both divisions were efficient in 2016.

As it was mentioned in the literature review some authors prefer to use the model under the constant returns to scale assumption (e.g. [2, 10, 16–18, 32, 37], or [41]), while others prefer variable returns to scale assumption (e.g. [11, 15, 20, 34], or [36]) presenting the advantages of both assumptions. As the contribution of this paper, we compare both approaches when variables are used in ratio form. We apply a non-parametric test for the equality of distributions presented by [81]. According to the density test results (T-statistics = 14.57; *p*-value = 2.22e-16), we can say that there are no significant differences between the DNDEA model under the constant returns to scale and variable returns to scale assumption. This way, we can verify the results of previous studies. In the case of ratio variables, the VRS model can be used as ratios can eliminate the size differences between the analysed countries.

We applied the input-oriented model to find out how the country could set its inputs effectively to reach given outputs. We analysed the optimal reduction in alcohol and tobacco consumption in the public health area, leading to given health-adjusted life expectancy in the specified country. The average alcohol consumption in 2000 was 9.475 l per capita aged 15 years and older, and tobacco consumption was 26.0806 per capita aged 15 years and over. The positive development could be seen as in 2016, the levels of indicators were only 8.9083 and 18.5722. The results pointed to the fact that there is still space for reduction of alcohol and tobacco consumption. According to the results at the given level of HALE, it is necessary to reduce the alcohol consumption by 6.85% and reduce the tobacco consumption by 4.59% on average. The results in the medical care area pointed to the fact that, on average, there is still room for more effective use of technologies and employees. The significant positive development in the equipment of the health sector could be seen during the analysed period. While in 2000, there were only 16.1 computer tomography devices per 1000,000 inhabitants, in 2016, the average number of devices was 25.9. Also, the number of employees increased from 10 doctors and nurses in health care calculated per 1000 inhabitants to 12.5. But it is important to say that a lower value of technologies and employees would also be sufficient at the given level of discharges and consultations. It indicates that after the period of increased investment into the technologies, it is also important to increase their effective usage in the form of an increased rate of their use in the diagnosis and treatment of the widest possible range of diseases. The positive impact of investment into the technologies and political decisions could be seen in the increasing number of screenings and increasing immunisation, and decreasing number of new cancer cases and decreasing infant mortality. The results of the analysis pointed to the fact that there is still space to improve also these indicators by a 0.27% increase in the case of immunisation, by 3% increase in the case of screenings, by 8% decrease in infant mortality and 3% decrease in new cancer cases (in average).

The DEA models are sensitive to extreme values (outliers) [54]. Thus, an analysis that focused on extreme values of inputs and outpust had been realised. The results demonstrated in this work may have implications for understanding the sensitivity of DEA models to outliers. With box-plot analysis, the number of OECD countries has reduced to 23 countries. The average efficiency increased by around 4% after the deviations were excluded. The presence of extremely low or extremely high inputs and outputs affect the efficiency score. We could speculate that this increase in efficiency could be due to a significant reduction in the research sample. The elimination of outliers led to a higher homogeneity of the results, which is confirmed by the lower standard deviation results. In 2016, variability decreased from 0.1057 to 0.0738.

By applying the Malmquist Index, the changes in the efficiency of countries over time were observed. In a comprehensive assessment, the performance in OECD countries in the health sector improved by 5% on average. The progress in the overall total factor productivity index was caused by the 16% growth in the relative technical efficiency (CU effect) and negative innovation (FS) effect by the 4%, which led to the shift of production possibility frontier.

Improvements in performance in several countries were mainly due to improvements in the public health sub-division (by 19%), while in the medical care sub-division, there was a decrease in performance (by 8%). The results of the Malmquist Index and its decompositions on the DNDEA model with the assumption of a CRS allowed identifying countries with performance growth in both sub-divisions, including Turkey, Korea, Germany, Luxembourg, the Netherlands and Greece. On the other hand, Slovakia, Poland, Chile, the United Kingdom, Lithuania, Norway, Latvia, Estonia and France recorded a decline in performance over the reporting period, mainly related to a decline in medical care sub-division. There was no decline in public health sub-division performance in any country during the reporting period.

The results of the Malmquist Index for the DNDEA model at VRS point to several changes in the ranking of countries. There has been an increase in the number of countries from 9 to 15, which achieved an overall decline in health performance in the reporting period, mainly due to a decline in the medical care sub-division.

Changes due to the CU effect point to an increase in performance in this area of 16% for OECD countries. In contrast, the average change due to the FS effect recorded a decrease of 4%. These results agree well with Dimas et al. [38] and Mitropoulos [41]. In their studies, these authors confirmed that the progression in productivity of OECD countries is related to a change in efficiency rather than a technological change.

This combination of findings supports the conceptual premise that gives us an interesting insight into the impact of individual factors on the efficiency of health systems in OECD countries and highlights the need for systematic development of a methodological platform for assessing health efficiency. National registers of individual countries are not sufficiently compatible to carry out comparative analyses as well as to examine the relationship of inputs and outputs within the health system.

The heterogeneity of countries’ health systems, their sensitivity to policy changes, the evolution of the demographic structure and their impact on pension systems, globalisation trends, migration, integration, political, economic and epidemiological crises will require a specific approach to exploring and identifying new factors affecting their effectiveness. This will also require a broader framework of international cooperation based on international networking with the development of mechanisms to eliminate inequalities in the health systems of the countries. Taken together, this remains an important priority for the health policies of OECD countries. This justifies the strong importance of national and international research studies to assess the effectiveness of health systems and puts pressure on the permanent, systematic development of methodological platforms and data systems in national and international registers.

## Conclusions

The efficiency of health systems is a long-term research domain of experts and professionals worldwide. Detecting resources of the inefficiency of the health system and defining optimal measures requires a long-term conceptual examination of all dimensions of the health system and strong cooperation between research teams and policymakers. In addition, changes in economies and many socio-economic and political impacts raise intriguing questions regarding the nature and extent of methodological platforms for assessing the efficiency of health systems.

In this study, the input-oriented DNDEA model with the assumption of a CRS and VRS was used to estimate the efficiency of health systems in OECD countries during 2000, 2008 and 2016. The overall health system efficiency for OECD countries under the assumption of CRS indicates that the average overall efficiency was 0.8801 in 2000, 0.8807 in 2008 and 0.8472 in 2016. The overall efficiency under the assumption of VRS indicates that the average efficiency was 0.9320 in 2000, 0.9504 in 2008 and 0.9371 in 2016. Comparing the results, we can say that the countries tend to be more efficient within the medical care sub-division than within the public health sub-division under both approaches.

By applying the Malmquist Index and its decompositions, significant changes in efficiency over time were observed. The 25 OECD countries have improved their performance levels over the reporting period. Partially, there was an improvement of 19% in the public health sub-division and a decrease of 8% in the medical care sub-division across countries with the assumption of constant returns to scale. Several other factors are also crucial in our analysis. On the one hand, it is a selection of inputs and outpust. In research studies, there are several inputs and outputs in the public health sub-division, and medical care sub-division, which may be used in a similar analysis using DNDEA models or classical DEA models. On the other hand, the analytical outputs represent a combination of conventional and unconventional methods of measuring health efficiency and provide many ideas for subsequent research in this field.

Despite the success demonstrated, a significant limitation of this study is the availability and complexity of databases. The inconsistency in the publication and updating of data partly limits the analytical possibilities and the subsequent development of methodologies. To realise multi-divisional analyses of the efficiency of health systems, it will be necessary to have access to deeper structured data and databases and ensure their interconnection at a national and international level.

Future trends are clear – health expenditure will increase in all OECD countries due to demographic ageing. This development will be determined by introducing advanced technologies (biotechnologies, nanotechnologies), innovative treatment practices and processes. However, a serious dilemma will be the long-term adjustment of the level of resources with the real costs and extent of healthcare.

As the number of older people increases, more elderly patients requiring specialised healthcare and long-term healthcare will need to be treated. Changes in the health behaviour and the current lifestyle of individuals support negative developments in the number of chronic diseases and avoidable mortality. From a macro-economic point of view, the serious question of the sustainability of the health and social system in the country is coming to mind.

This also creates intense pressures for the further development of methodologies that would examine efficiency in the health sector and reveal its other determinants. Also, it would support the development of international benchmarks in this area and encourage the implementation of international comparative analyses. These are essential for properly setting health policies in countries and drafting regional and national health sustainable plans. Tackling this issue requires an ongoing performance of comparative analyses and active policy concepts to eliminate health inequalities. It is not possible to evaluate them without performing high-quality comparative analyses of the efficiency of health systems in countries and creating international benchmarks.

## Data Availability

The health-adjusted life expectancy (HALE) was collected from the Global Health Observatory database, which is World Health Organization’s data repository for health-related statistics for its all member states (WHO is United Nations specialised agency for public health and it is headquartered in Geneva, Switzerland). The HALE can be found under the section of „Indicator Groups “in the sub-section „Healthy life expectancy“. All other indicators were collected from the OECD. Stat statistical online platform of the OECD’s statistical databases (OECD stands for Organisation for Economic Co-operation and Development, and it is headquartered in Paris, France). The Alcohol consumption and Tobacco consumption are available in the section „Health “and in the sub-section „Non-Medical Determinants of Health“. Immunisation, Breast cancer screening and Cervical cancer screening are available in the section „Health “in the sub-section „Health Care Utilisation “in the dimensions „Immunisation “or „Screening“, respectively. Infant mortality and Incidence of cancer are available in the section „Health “in the sub-section „Health Status “in the dimensions „Maternal and infant mortality “or „Cancer“, respectively. Medical technology refers to the number of computer tomography devices per one million inhabitants, and it is available in the section „Health “in the sub-section „Health Care Resources“. Employment in healthcare is available in the section „Health “in „Health Care Resources “in the dimension „Total health and social employment“. Inpatient discharges are available in the section „Health “in the sub-section „Health Care Utilisation “in the dimension „Hospital discharges by diagnostic categories“. Consultations are available in the section „Health “in the sub-section „Health Care Utilisation “and refer to the average number of consultations per physician per capita.

## Abbreviations

AUGE: Universal Access to Explicit Guaranteed Entitlements
AUS: Australia
AUT: Austria
Avg: Average
BCC: Banker, Charnes and Cooper model
BEL: Belgium
CAN: Canada
CCR: Charnes, Cooper and Rhodes model
CO: carry-over variable
CRS: Constant Returns to Scale
CU: Catch-Up effect
CZE: Czech Republic
DEA: Data Envelopment Analysis
DEU: Germany
DMU: Decision Making Unit
DNDEA: Dynamic Network Data Envelopment Analysis
DNK: Denmark
EC: Efficiency Change
ESP: Spain
EST: Estonia
FDH: Free Disposal Hull
FIN: Finland
FONASA: Fondo Nacional de Salud
FRA: France
FS: Frontier Shift effect
GBR: United Kingdom
GDP: Gross Domestic Product
GRC: Greece
HALE: Health-Adjusted Life Expectancy
HIV/AIDS: Human Immunodeficiency Virus infection and Acquired Immune Deficiency Syndrome
HUN: Hungary
CHE: Switzerland
CHL: Chile
IRL: Ireland
ISL: Iceland
ISR: Israel
ITA: Italy
JPN: Japan
KOR: Korea
LE: Life Expectancy at birth
LTU: Lithuania
LUX: Luxembourg
LVA: Latvia
Max: Maximum
MC: Medical Care division
MEX: Mexico
MI: Malmquist Index
Min: Minimum
NLD: Netherlands
NOR: Norway
NZL: New Zealand
OECD: Organisation for Economic Co-operation and Development
PH: Public Health division
POL: Poland
PPP: Purchasing Power Parity
PRT: Portugal
QALY: Quality-Adjusted Life Years
SFA: Stochastic Frontier Analysis
SVK: Slovakia
SVN: Slovenia
SWE: Sweden
TC: Technology Change
TUR: Turkey
US$: United States dollar
USA: United States
VRS: Variable Returns to Scale
WHO: World Health Organization
